# Gut microbial community structure, metabolic signature, and resistome in dyslipidemia: implications for cardiovascular disease management

**DOI:** 10.1128/spectrum.00971-25

**Published:** 2026-03-04

**Authors:** Soomin Lee, Hyung-Lae Kim, Shahbaz Raza, Eun-Ju Lee, Yoosoo Chang, Seungho Ryu, Juhee Cho, Han-Na Kim

**Affiliations:** 1 Department of Clinical Research Design and Evaluation, Samsung Advanced Institute for Health Sciences and Technology, Sungkyunkwan University 35017, , Seoul, Republic of Korea; 2 Department of Biochemistry, College of Medicine, Ewha Womans University 26717, Seoul, Republic of Korea; 3 Center for Cohort Studies, Total Healthcare Center, Kangbuk Samsung Hospital, Sungkyunkwan University School of Medicine 35019, Seoul, Republic of Korea; 4 Department of Occupational and Environmental Medicine, Kangbuk Samsung Hospital, Sungkyunkwan University School of Medicine 35019, Seoul, Republic of Korea; 5 Center for Clinical Epidemiology, Samsung Medical Center, Sungkyunkwan University 35017, , Seoul, Republic of Korea; 6 Departments of Epidemiology and Medicine, and Welch Center for Prevention, Epidemiology and Clinical Research, John Hopkins Medical Institutions, Baltimore, Maryland, USA; Nova Southeastern University, Fort Lauderdale, Florida, USA

**Keywords:** dyslipidemia, gut microbiome, shotgun metagenomics, functional profiling, cardiovascular disease

## Abstract

Dyslipidemia, characterized by abnormal blood lipid levels, constitutes a significant risk factor for cardiovascular disease. Emerging evidence indicates that the gut microbiota influences lipid metabolism, although findings across studies have been inconsistent. In this cross-sectional investigation, we analyzed the composition of gut microbiota, associated metabolic pathways, predicted gut metabolites, and the resistome in 1,384 participants (including 895 individuals with dyslipidemia and 489 controls) through shotgun metagenomic sequencing. Our findings demonstrated that *Bacteroides caccae* was enriched among dyslipidemia cases, potentially contributing to inflammation and altered lipid metabolism. Conversely, *Coprococcus eutactus* and *Coprococcus catus*, recognized producers of short-chain fatty acids (SCFAs) involved in lipid regulation, as well as *Blautia obeum*, known to be positively affected by SCFAs, were more prevalent in the control group. Additionally, we identified an enrichment of the gene family responsible for dTDP-beta-D-fucofuranose biosynthesis, associated with bacterial pathogenicity, in dyslipidemia cases, with *Bacteroides stercoris* serving as a major contributor. Dyslipidemia cases also exhibited depletion of glycogen and peptidoglycan biosynthesis pathways, which may compromise energy storage and immune function, alongside decreased levels of pseudouridine, a molecule involved in RNA metabolism. Furthermore, a marginal increase in abundance of antibiotic-resistance genes, *tetQ*, was observed in dyslipidemia cases, suggesting a potential link between the gut resistome and metabolic dysregulation. These results offer novel insights into the role of gut microbiota in the pathophysiology of dyslipidemia and underscore potential microbiome-targeted interventions for metabolic disease management.

**IMPORTANCE:**

Dyslipidemia, characterized by abnormal blood lipid levels, is a significant risk factor for cardiovascular disease. Emerging evidence suggests that the gut microbiota plays a role in lipid metabolism, although findings across studies have varied. This study analyzed the gut microbiota, metabolic pathways, predicted gut metabolites, and antimicrobial resistance genes in 1,384 participants using shotgun metagenomic sequencing. Individuals with dyslipidemia exhibited an imbalance in gut bacteria, including an increase in *Bacteroides caccae*, a species associated with inflammation, and a decrease in short-chain fatty acid-producing bacteria such as *Coprococcus eutactus* and *Blautia obeum*, which support metabolic health. Furthermore, we identified significant changes in microbial metabolic pathways related to energy storage and immune function, as well as an increased abundance of tetracycline resistance genes (*tetQ*), suggesting a potential link between dyslipidemia and antimicrobial resistance. Our study provides a comprehensive overview of dyslipidemia-associated gut microbial alterations, highlighting potential mechanistic links and therapeutic targets.

## INTRODUCTION

Dyslipidemia, characterized by abnormal blood lipid levels, constitutes a significant risk factor for cardiovascular disease (CVD). According to the 2020 disease statistics of Korea, the prevalence of dyslipidemia was 45.6% among men and 31.3% among women (1), with its incidence increasing notably, particularly among younger individuals, including those with a normal body mass index (BMI). Both genetic and environmental factors contribute to its etiology, and while lifestyle modifications can mitigate associated risks, the fundamental biological mechanisms underlying it remain only partially understood.

Recent research indicates that the gut microbiota may play a crucial role in lipid metabolism and the development of dyslipidemia. Observational studies have established links between specific gut microbial profiles and lipid biomarkers, showing associations between gut bacterial genera and blood lipid levels (2–5). For example, *Prevotella* and *Bacteroides* have been associated with lipid metabolism in men, while *Akkermansia* and *Escherichia*/*Shigella* have been implicated in women (5). More recently, Mendelian Randomization studies have provided evidence of a potential causal relationship between gut microbiota and dyslipidemia (6, 7), strengthening the hypothesis that microbial composition influences host lipid metabolism. Despite these findings, there remain several limitations in current microbiome research related to dyslipidemia. Many investigations have relied on 16S rRNA sequencing, which offers only genus-level taxonomic resolution and limited functional insights into microbial metabolism. A comprehensive, functional characterization of the gut microbiome in dyslipidemia through shotgun metagenomic sequencing remains largely unexplored.

Beyond taxonomic composition, the gut microbiota influences lipid metabolism through microbial metabolites. Individuals with dyslipidemia show metabolic changes, including low levels of short-chain fatty acids (SCFAs) and high levels of pro-inflammatory molecules like lipopolysaccharides (LPS) and trimethylamine N-oxide (TMAO) (8). Notably, TMAO, a metabolite derived from dietary choline and phosphatidylcholine by gut bacteria, has been associated with an increased risk of cardiovascular disease, potentially through effects on lipid absorption and cholesterol metabolism (9, 10). However, the specific metabolic pathways involved in dyslipidemia remain insufficiently understood.

An emerging yet understudied factor in metabolic diseases is the gut resistome, the collection of antimicrobial resistance genes (ARGs) within the gut microbiome. Although increased ARGs have been associated with inflammatory bowel disease (11), metabolic disorders (12, 13), and even cancer (14), their role in dyslipidemia remains ambiguous. Previous research has indicated that antibiotic residues such as ciprofloxacin, trimethoprim, and tetracycline may contribute to dyslipidemia by altering gut microbial composition and function (15, 16). Given the extensive use of antibiotics in both clinical and agricultural contexts, examining the gut resistome in relation to dyslipidemia is crucial for gaining a comprehensive understanding of broader microbiome-mediated metabolic disturbances.

To address these knowledge gaps, we conducted a large-scale shotgun metagenomic analysis on a well-characterized Korean cohort (*n* = 1,384) to explore the role of the gut microbiome in dyslipidemia. Our study aims to identify microbial species linked to dyslipidemia, clarify the functional capabilities of the gut microbiota, including metabolic pathways and predicted metabolite changes, and characterize gut resistome profiles and their potential effects on lipid metabolism and host health. Using shotgun metagenomics, this study offers a detailed taxonomic and functional overview of the gut microbiome in dyslipidemia, providing new insights into microbial signatures, metabolic pathways, and antibiotic resistance genes associated with the condition.

## RESULTS

### Characteristics of subjects and their sequencing data

We analyzed shotgun metagenomic sequencing data to investigate the gut microbiome in subjects with dyslipidemia comprehensively. After removing low-quality reads and filtering out host (human) DNA reads, we retained high-quality microbial sequencing data with an average of 6.36 gigabases (Gb) and 42.4 million reads per sample, which were used for subsequent taxonomic and functional analyses.

Among 1,384 participants (895 dyslipidemia cases and 489 controls, Fig. S1), male participants showed a significantly higher prevalence of dyslipidemia (*P* < 0.05, chi-squared test, Table 1). Dyslipidemia cases were also notably older and had higher BMI compared to controls. Furthermore, they displayed higher blood glucose levels, systolic blood pressure (SBP), and diastolic blood pressure (DBP) than the control group (*P* < 0.05, *t*-test). Due to the strong correlations among variables, we checked for multicollinearity and only included independent variables as covariates in further analyses. We adjusted for sex, age, BMI, blood glucose, and SBP but excluded DBP to prevent collinearity.

**TABLE 1:** Study participant characteristics^*b*^

Variables	Overall (*n* = 1,384)	Dyslipidemia (*n* = 895)	Control (*n* = 489)	*P*-value^*a*^
Sex				<0.001
Male	931 (67.3%)	648 (72.4%)	283 (57.9%)	
Female	453 (32.7%)	247 (27.6%)	206 (42.1%)	
Age (years), mean ± SD	45.54 ± 8.38	46.3 ± 8.2	44.2 ± 8.5	<0.001
BMI (kg/m), mean ± SD	24 ± 3	24.7 ± 3.2	22.9 ± 2.9	<0.001
Blood glucose level (mg/dL) mean ± SD	95.9 ± 13.8	97.6 ± 15.7	92.8 ± 8.4	<0.001
Total cholesterol (mg/dL), mean ± SD	198 ± 33	212.8 ± 30.2	171.9 ± 18.6	<0.001
TG (mg/dL), mean ± SD	121 ± 72	143.2 ± 78.9	81.6 ± 29.0	<0.001
HDL-C (mg/dL), mean ± SD	57 ± 14	54.0 ± 14.2	61.5 ± 12.5	<0.001
LDL-C (mg/dL), mean ± SD	125 ± 31	138.9 ± 27.2	98.9 ± 18.3	<0.001
SBP (mmHg), mean ± SD	111 ± 12.9	112.8 ± 12.7	106.9 ± 12.4	<0.001
DBP (mmHg), mean ± SD	72 ± 9.7	73.4 ± 9.6	69.1 ± 9.3	<0.001
Alcohol (grams), mean ± SD	5 ± 4	16.3 ± 26.7	14.3 ± 20.9	0.29
No response (*n*, %)	480 (34.7%)	342 (38.2%)	138 (28.2%)	
Smoker (%)				<0.001
Smoker	176 (12.7%)	128 (23.1%)	48 (13.5%)	
Non-smoker	734 (53.0%)	426 (76.9%)	308 (86.5%)	
No response (*n*, %)	474 (34.2%)	341 (38.1%)	133 (27.2%)	
Exercise (%)				0.85
None	533 (38.5%)	331 (57.4%)	202 (55.5%)	
<3 d	261 (18.9%)	157 (27.2%)	104 (28.6%)	
≥3 d	147 (10.6%)	89 (15.4%)	58 (15.9%)	
No response (*n*, %)	443 (32.0%)	318 (35.5%)	125 (25.6%)	
FFQ energy (kcal/day), mean ± SD	1,422 ± 621	1,390.3 ± 615.0	1,471.3 ± 628.4	0.094
No response (*n*, %)	687 (49.6%)	467 (52.2%)	220 (45.0%)	
FFQ total protein (g/day), mean ± SD	49 ± 24	48.4 ± 24.3	50.2 ± 24.5	0.325
No response (*n*, %)	687 (49.6%)	467 (52.2%)	220 (45.0%)	
FFQ total fat (g/day), mean ± SD	29 ± 19	28.1 ± 18.1	29.8 ± 19.8	0.231
No response (*n*, %)	687 (49.6%)	467 (52.2%)	220 (45.0%)	
FFQ carbohydrate (g/day), mean ± SD	238 ± 107	232.4 ± 105.4	247.1 ± 108.6	0.078
No response (*n*, %)	687 (49.6%)	467 (52.2%)	220 (45.0%)	

^*a*^ *P*-value was estimated by *t*-test for continuous variables and the chi-squared test for categorical variables.

^*b*^ BMI, body mass index; TG, triglycerides; HDL-C, high-density lipoprotein cholesterol; LDL-C, low-density lipoprotein cholesterol; SBP, systolic blood pressure; DBP, diastolic blood pressure; d, days; FFQ, food frequency questionnaire; SD, standard deviation.

### Microbial diversity and differential abundance analysis in dyslipidemia

To examine the differences in microbial community structure between the dyslipidemia group (*n* = 895) and the control group (*n* = 489), we first examined alpha diversity. In unadjusted models, dyslipidemia was associated with lower observed richness, Simpson, Shannon, and Pielou’s evenness (Fig. S2), but these differences were attenuated after covariate adjustment (Table S1). Sex and BMI showed independent associations with all alpha diversity indices, and a dyslipidemia × BMI interaction was observed for several metrics (Table S1). In lipid trait-specific analyses, hypertriglyceridemia showed association with reduced alpha diversity with and without covariate adjustments (Table S2). Dyslipidemia cases without hypertriglyceridemia showed reduced alpha diversity only in unadjusted analyses.

For beta diversity, differences in overall microbial community structure between dyslipidemia cases and controls were assessed using multiple distance metrics. Before covariate adjustment, all five beta diversity indices showed statistically significant differences between groups (*P* < 0.05); however, these associations were attenuated after accounting for host factors. In covariate-adjusted pairwise permutational multivariate analysis of variance (PERMANOVA) models, only the Aitchison distance showed a statistically significant difference between groups (R² = 0.0012, *P* < 0.01), whereas Bray–Curtis, Jaccard, weighted UniFrac, and unweighted UniFrac distances were not significant (*P* > 0.05). Consistent with the small effect size observed in the adjusted model, principal coordinate analysis (PCoA) plots did not show clear visual separation between dyslipidemia cases and controls (Fig. S3).

To identify bacterial species associated with dyslipidemia, we analyzed species-level relative abundance across all participants. A total of 755 bacterial species were detected, of which 171 species present in more than 10% of individuals were included in downstream analyses. Among all participants, *Prevotella copri* (19.28%) and *Bacteroides plebeius* (7.52%) were the most prevalent species. In unadjusted Microbiome Multivariate Association with Linear Models 2 (MaAsLin2) (17) models, no species showed significant differences between dyslipidemia and control groups (*q* > 0.05). In contrast, after covariate adjustment, dyslipidemia was associated with higher abundances of *Bacteroides caccae* and lower abundances of *Coprococcus eutactus*, *Coprococcus catus*, and *Blautia obeum* (*q* < 0.05, Table 2; Fig. S4). Three additional species, including *Bacteroides stercoris*, *Roseburia inulinivorans,* and *Dorea longicatena*, showed marginal associations (0.05 ≤ *q* < 0.1; Table S3).

**TABLE 2:** Differentially abundant bacterial species in dyslipidemia cases and controls^*d*^^,^^*e*^

Bacterial species	Abundance (%)^*a*^		MaAsLin2^*b*^				ANCOM-BC^*c*^	
	Dyslipidemia (*n* = 895)	Control (*n* = 489)	Coefficient (SE)	Number of not 0	*P*-value	*q*-value	Log-fold change (SE)	*P*-value
*Coprococcus eutactus*	0.42	0.55	−1.11 (0.33)	597	0.001^**^	0.008^**^	−0.07 (0.03)	0.011^*^
*Coprococcus catus*	0.05	0.31	−0.69 (0.24)	1053	0.004^**^	0.033^*^	−0.01 (0.005)	0.008^**^
*Bacteroides caccae*	0.84	0.41	0.95 (0.33)	986	0.004^**^	0.034^*^	0.04 (0.03)	0.241
*Blautia obeum*	0.12	0.25	−0.6 (0.22)	1185	0.006^**^	0.044^*^	−0.02 (0.01)	0.146

^*a*^ Average relative abundance (%) of bacterial species in dyslipidemia case and control.

^*b*^ The coefficient and* p*-values were calculated by adjusting for covariates using the generalized linear model implemented in Multivariate Association with Linear Models (MaAsLin2). The *q*-values were calculated using the Benjamini-Hochberg method for multiple testing correction.

^*c*^ Log-fold changes were obtained from the Analysis of Compositions of Microbiomes with Bias Correction program (ANCOM-BC) log-linear model. The *p*-values were calculated from a two-sided Z-test.

^*d*^ ^* ^*P* < 0.05, ^** ^*P* < 0.01. Abbreviation. SE, standard error.

^*e*^ Adjusted for age, sex, BMI, blood glucose levels, and SBP.

To assess robustness, we conducted complementary analyses using Analysis of Compositions of Microbiomes with Bias Correction (ANCOM-BC). Among the four species identified in the primary MaAsLin2 analysis, *C. eutactus* and *C. catus* were also significant in ANCOM-BC (*P* < 0.05), while all three species showing marginal associations in MaAsLin2 (*B. stercoris*, *R. inulinivorans*, and *D. longicatena*) were confirmed as significant by ANCOM-BC (*P* < 0.05; Table S3).

Additional exploratory analyses were performed to evaluate the consistency of these associations across host subgroups and lipid traits. In stratified analysis by age, BMI, SBP, and blood glucose level, the four bacterial species identified in the primary analysis showed consistent effect direction and comparable effect sizes in higher strata of age, BMI, SBP, and blood glucose levels, whereas statistical significance was attenuated in lower strata (Table S4). In lipid trait-specific analyses, *C. eutactus* was significantly depleted in hypertriglyceridemia and elevated LDL-C, while *C. catus* and *B. obeum* were significantly depleted in hypertriglyceridemia (*P* < 0.05, Table S5). Additionally, all four differentially abundant taxa remained significantly associated in dyslipidemia cases without hypertriglyceridemia. For the other individual lipid traits, associations showed similar directional trends but did not reach statistical significance (*P* > 0.05). These stratified and trait-specific analyses were considered supportive and exploratory, and the covariate-adjusted MaAsLin2 results were treated as the primary species-level findings.

### Identification of metabolic pathways and bacterial species contributing to the pathways in dyslipidemia

**Fig 1:**
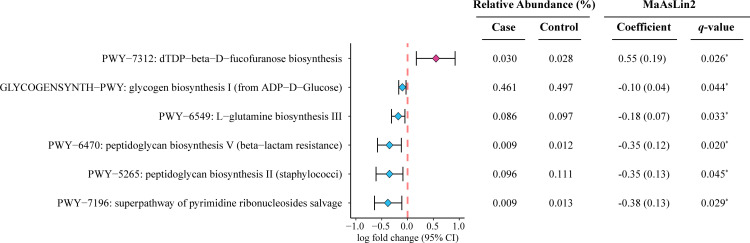
Differentially abundant metabolic pathways between dyslipidemia (*n* = 895) and control (*n* = 489) groups. The coefficient and *p*-values were calculated using the linear regression model implemented in the Microbiome Multivariable Association with Linear Models, adjusting for five covariates, including age, sex, body mass index (BMI), blood glucose levels, and systolic blood pressure (SBP). The *q*-values were calculated using the Benjamini-Hochberg method for multiple testing corrections. ^*^q < 0.05.

To investigate the functional role of the gut microbiome in dyslipidemia, a differential abundance analysis of metabolic pathways was conducted using shotgun sequencing data from all participants. Initially, 14 metabolic pathways demonstrated differential abundance between the dyslipidemia and control groups, without adjustment for covariates (Table S6). Following covariate adjustment, six pathways remained significantly different (*q* < 0.05). Notably, only the dTDP-beta-D-fucofuranose biosynthesis pathway (PWY-7312) was found to be enriched in the dyslipidemia group (Fig. 1). The remaining five pathways, including glycogen biosynthesis (GLYCOGENSYNTH-PWY), L-glutamine biosynthesis (PWY-6549), peptidoglycan biosynthesis (PWY-6470 and PWY-5265), and the superpathway of pyrimidine ribonucleoside salvage (PWY-7196), were more enriched in the control group. Furthermore, five metabolic pathways exhibited marginal differences between the groups (*q* < 0.1), namely the pentose phosphate pathway (NONOXIPENT-PWY, coefficient: –0.096, *q* = 0.054, *P* < 0.05), peptidoglycan biosynthesis IV (PWY-6471, coefficient = –0.256, *q* = 0.058, *P* < 0.05), ppGpp metabolism (PPGPPMET-PWY, coefficient = –0.213, *q* = 0.077, *P* < 0.05), ADP-L-glycero-beta-D-manno-heptose biosynthesis (PWY-1241, coefficient = 0.149, *q* = 0.079, *P* < 0.05), and L-ornithine biosynthesis II (ARGININE-SYN4-PWY, coefficient = 0.192, *q* = 0.083, *P* < 0.05). These findings are detailed in Table S7.

To quantify the functional contributions of bacterial communities, we used the HUMAnN3 framework and stratified these functions based on the contributing species that contributed, called the “contributional diversity” of the function (18). Pathways with contributions from multiple species showed high within-sample (“complex”) and high between-sample (“variable”) contributional diversity (18). For the dTDP-beta-D-fucofuranose biosynthesis pathway (PWY-7312), *B. stercoris* and *B. clarus* were identified as the main contributors (Fig. S5A). In the glycogen biosynthesis I pathway (GLYCOGENSYNTH-PWY), *Faecalibacterium prausnitzii*, *Escherichia coli*, and *Anaerostipes hadrus* were the top three contributors (Fig. S5B; Table S8). For L-glutamine biosynthesis III (PWY-6549), only unclassified species were identified as contributors (Fig. S5C). Additionally, for the peptidoglycan biosynthesis pathway (PWY-5265 and PWY-6470), *Enterococcus faecium*, *Lactobacillus fermentum*, and various *Streptococcus* species, including *S. lutetiensis*, were the main contributing bacterial species (Fig. S5D and E). The superpathway of pyrimidine ribonucleosides salvage (PWY-7196) lacked enough species contribution information to generate reliable bar plots.

### Predicted gut metabolites and their association with bacterial species and pathways

Using the UniRef90 gene profiles obtained from HUMAnN3, we predicted gut metabolites with MelonnPan (19) and identified a total of 80 metabolites across all individuals. Among them, 42 metabolites were significantly different between the dyslipidemia and control groups before adjusting for covariates (*q* < 0.05, MaAsLin2). Metabolites such as C2 carnitine, C16 carnitine, ketodeoxycholate, cholate, and chenodeoxycholate were enriched in the dyslipidemia group, while undecanedionate, urobilin, and lithocholic acid were more abundant in the control group (Table S9). After adjusting for covariates, pseudouridine remained significantly depleted in the dyslipidemia group (coefficient: −0.026, *q* < 0.05, Table S8). Additionally, uracil (coefficient = −0.026, *q* = 0.052, *P* < 0.05), N-acetylhistidine (coefficient = −0.010, *q* = 0.071, *P* < 0.05), and ketodeoxycholate (coefficient = 0.100, *q* = 0.070, *P* < 0.05) showed marginal differences between groups (*q* < 0.1, Table S10).

**Fig 2:**
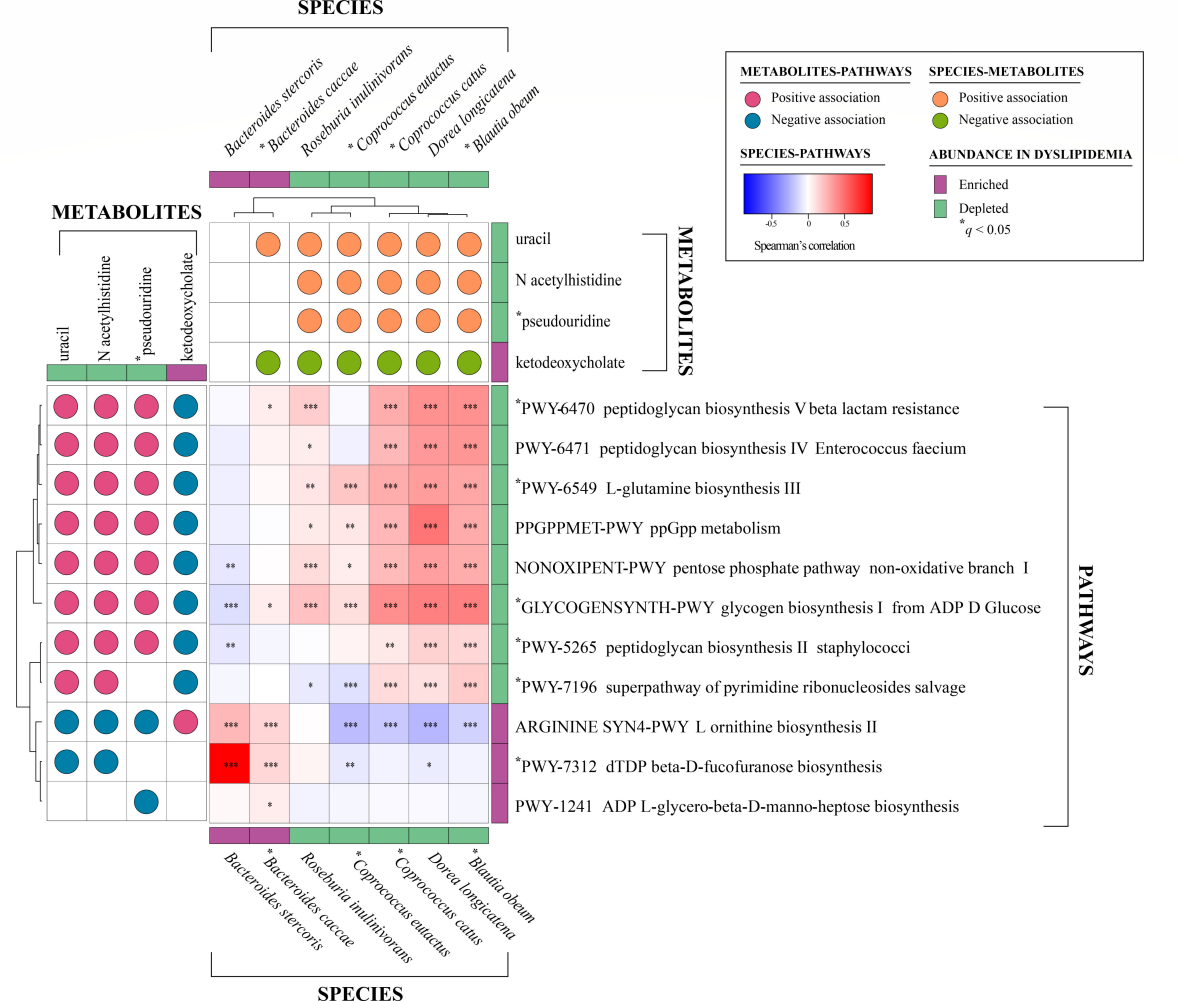
The tripartite correlation heatmap of differentially abundant microbial species, metabolic pathways, and predicted gut metabolites for dyslipidemia (*n* = 895) and control (*n* = 489) groups. Right panel denotes the correlations between species and metabolic pathways. The top panel denotes the correlations between metabolic pathways and predicted gut metabolites. The left panel denotes the correlations between species and predicted gut metabolites. The correlation coefficients and *P*-values were calculated using Spearman’s correlation test. The *P*-values were adjusted using the Benjamini-Hochberg multiple testing correction. ^*^q <0.05, ^**^q <0.01, ^***^q <0.001.

To explore the integration of functional and metabolite-level signals, we examined correlations between significantly altered pathways and predicted metabolites, including species-level associations (Fig. 2). Of the six pathways significantly changed after covariate adjustment, three, including peptidoglycan biosynthesis (PWY-6470 and PWY-5265) and glycogen biosynthesis (GLYCOGENSYNTH-PWY), showed positive correlations with uracil, N-acetylhistidine, and pseudouridine, all of which were predicted to be depleted in the dyslipidemia group. These pathways were also positively associated with *C. catus* and *B. obeum* (species enriched in controls) and negatively with ketodeoxycholate, a bile acid metabolite elevated in dyslipidemia. Additionally, PWY-7196 (superpathway of pyrimidine ribonucleosides salvage) includes uracil as a direct biochemical component and showed consistent reductions at both the pathway and metabolite levels. For other pathways, such as dTDP-beta-D-fucofuranose biosynthesis (PWY-7312), no directly mapped metabolite was found in the prediction set. However, this pathway showed positive correlation with *B. stercoris* and *B. caccae*, and negative correlation with *C. eutactus*, *D. longicatena*, uracil, and N-acetylhistidine. Ketodeoxycholate exhibited negative correlations with most species except *B. stercoris*, while uracil, N-acetylhistidine, and pseudouridine were positively correlated with species depleted in dyslipidemia.

### Antibiotic-resistance genes (ARGs) and virulence factor genes (VFGs) in dyslipidemia and their associations with gut microbiome

**Fig 3:**
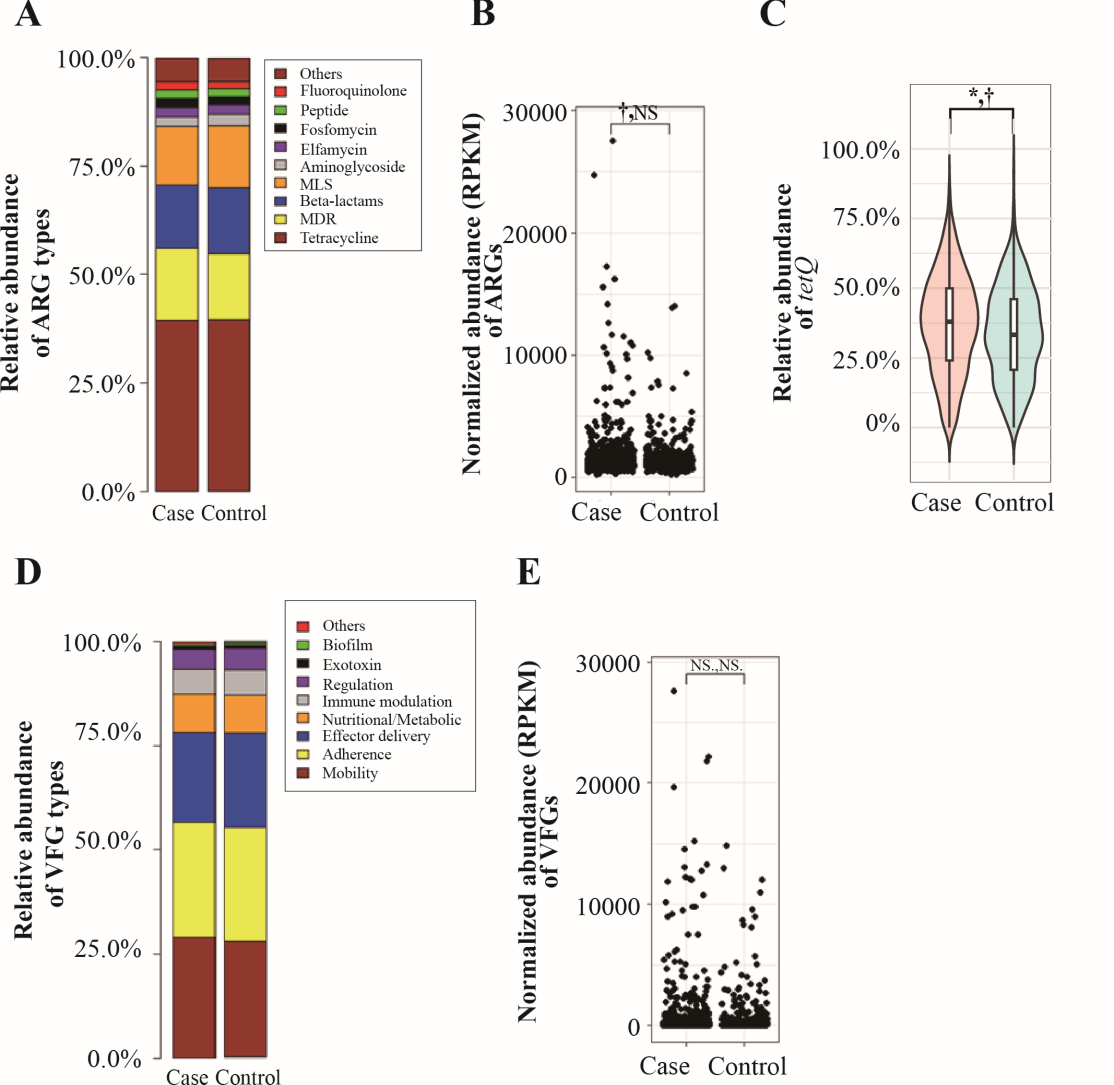
Gut resistome and virulence factor gene (VFGs) composition of healthy individuals (*n* = 489) and those with dyslipidemia (*n* = 895). (**A**) Stacked bar plot of total sum scaling (TSS)-normalized relative abundance of antibiotic resistance genes (ARGs) classes, (**B**) jitter plot for differential abundance analysis of total ARG abundance based on reads per kilobase of transcript per million reads mapped (RPKM) between groups using linear regression models adjusted for five covariates, (**C**) box plot for TSS-normalized relative abundance of differentially abundant *tetQ* gene With corrected *P*-values, (**D**) stacked bar plot of TSS-normalized relative abundance of virulence factor genes (VFGs) classes, (**E**) and jitter plot for differential abundance analysis of VFGs based on RPKM between both groups using linear regression models adjusted for covariates (**E**). Comparative analyses of differential abundance were performed using linear regression models implemented in Multivariate Association with Linear Models (MaAsLin2) with and without adjusting for five covariates, including age, sex, body mass index, systolic blood pressure, and fasting glucose level. ^*^P < 0.05, ^*^q < 0.05, ^†^ < 0.1, NS, non-significant.

By investigating the presence and distribution of ARGs and VFGs across the gut microbiome samples in all individuals, we identified 384 different ARG subtypes across the gut microbiome samples, belonging to 17 ARG types, in all individuals. For downstream analysis, we selected 127 ARG subtypes present in >10% of the samples. The most abundant ARG types were tetracycline, followed by multidrug resistance, beta-lactams, and macrolide-lincosamide-streptogramin (MLS) in all subjects (Fig. 3A). Before adjusting covariates, total ARG prevalence was marginally higher in the dyslipidemia group compared to controls (*P* < 0.1, Fig. 3B). However, this marginal significance disappeared after covariate adjustment.

Alpha diversity indices for ARG subtypes, including Simpson index, Pielou’s evenness, and Shannon entropy, were significantly lower in dyslipidemia (*n* = 895) than in the control group (*n* = 489), before adjusting for covariates (*P* < 0.05) (Fig. S6). After adjustments, only the Simpson index (coefficient = −0.019) and Pielou’s evenness (coefficient = −0.02) indices remained significantly lower in the dyslipidemia group (*P* < 0.05). For beta diversity, significant differences between dyslipidemia and control groups were observed for Bray-Curtis dissimilarity (R^2^ = 0.0034, PERMANOVA) and Jaccard dissimilarity (R^2^ = 0.0027) without adjusting covariates (*P* < 0.01). However, no significant difference was observed for Aitchison distance (*P* > 0.05). After adjusting for covariates, Bray-Curtis dissimilarity (R^2^ = 0.0016) and Jaccard dissimilarity (R^2^ = 0.0013) remained significant (*P* < 0.05), but Aitchison distance did not. Despite these differences, no clear separation between groups was visible in PCoA plots (Fig. S7).

Differential abundance analysis for the ARGs at the class level showed no significant differences between dyslipidemia and control groups, regardless of covariate adjustment. At the subtype level, the most common ARG subtypes across all subjects were *tetQ, cfxA, ermF, ermG, tetW, tetO*, and *ermB* (Table S11). Before covariate adjustment, the relative abundance of *tetQ* (coefficient = 0.035, *q* < 0.05) was significantly higher in dyslipidemia cases than in controls, while other subtypes showed no significant differences (Fig. 3C). After adjustment, *tetQ* remained marginally more abundant in dyslipidemia cases (coefficient = 0.029, *P* < 0.05, *q* = 0.087). To assess robustness, we conducted an internal validation using repeated stratified 70/30 train-test splits. The direction and magnitude of the dyslipidemia-*tetQ* association were consistent across the training and test sets, although statistical significance was mainly retained in the training sets and attenuated in the smaller test subsets (Table S12). Bootstrap resampling analyses (1,000 iterations) further indicated a stable distribution of effect estimates with a 99.7% of positive direction (Table S13).

Further correlation analysis indicated that *tetQ* correlates with dyslipidemia-associated species; it was positively correlated with *B. stercoris* and negatively correlated with *C. eutactus*, *C. catus*, *B. obeum, R. inulinivorans,* and *D. longicatena* (*P* < 0.05, Table S14).

Analysis of food frequency questionnaire (FFQ) data from 889 individuals (566 dyslipidemia cases and 323 controls) revealed that those who consumed pork at least 1–2 times per week (*n* = 228) had significantly higher abundance of *tetQ* than those with less frequent pork consumption (*n* = 661; coefficient = 60.1, *P* = 0.022, after adjustment for five covariates, linear regression model). In contrast, individuals consuming beef more than once per month (*n* = 730) showed a non-significant association with *tetQ* abundance (*n* = 159; coefficient = 3.3, *P* = 0.913 after adjustment for five covariates). Logistic regression analysis further showed that dyslipidemia was not significantly associated with the proportion of pork consumers at least 1–2 times per week (*P* > 0.05, logistic regression model), whereas a significant association was observed with the proportion of beef consumers more than once per month (odds ratio = 1.45, *P* < 0.05), after adjustment for five covariates. Furthermore, we additionally tested frequency cutoffs for meat consumption: more than once per month for pork and more than once per week for beef. These supplementary analyses revealed no significant associations between meat consumption and either *tetQ* abundance or dyslipidemia prevalence (*P* > 0.05, after adjustment for five covariates, Table S15).

We observed that VFGs related to motility, adherence, and effector delivery systems were the most common across all individual samples (Fig. 3D). Although the overall abundance of VFGs appeared higher in the dyslipidemia group, no significant differences were observed between groups (*P* > 0.05, linear regression model, Fig. 3E). Additionally, no individual VFG types and subtypes showed significant differences between dyslipidemia cases and controls before and after adjusting for covariates (*q* > 0.05).

In the correlation analysis between the abundance of ARGs/VFGs and phenotypic factors, including the total cholesterol (TC), triglycerides (TG), low-density lipoprotein cholesterol (LDL-C), high-density lipoprotein cholesterol (HDL-C), blood glucose levels, BMI, age, SBP, and DBP in all individuals, a significant positive correlation was observed between tetracycline-type ARGs and LDL-C (coefficient = 0.092), SBP (coefficient = 0.078), and DBP (coefficient = 0.082, *q* < 0.05, Fig. S8). Tetracycline-type ARGs also showed a negative correlation with most ARGs and VFGs, with *tetQ* demonstrating similar correlation patterns. In contrast, other ARG types, such as beta-lactams, showed strong positive correlations with most VFGs. Adherence-associated VFs were positively correlated with most phenotypes, except for TC, HDL-C, and age. Among demographic phenotypic factors, blood glucose levels, BMI, and SBP were negatively correlated with most ARGs and VFGs.

## DISCUSSION

In this study, we investigated associations between gut microbiome composition, functional potential, resistome, and dyslipidemia using shotgun metagenomic sequencing. After rigorous covariate adjustment, we identified dyslipidemia-associated differences primarily at the level of specific bacterial taxa and functional pathways, whereas global diversity metrics were largely explained by host factors. These findings suggest that dyslipidemia is associated with targeted alterations in microbial composition and metabolic capacity rather than broad community-wide shifts.

A central finding was the consistent depletion of SCFA-producing bacteria, including *C. eutactus, C. catus*, and *B. obeum*, in the dyslipidemia group. These species are known to generate SCFAs (20) such as acetate, propionate, and butyrate, which are essential for maintaining intestinal barrier integrity, modulating systemic inflammation, and regulating host lipid metabolism (21). Reduced SCFA production has been associated with increased gut permeability and systemic endotoxemia, which may promote chronic low-grade inflammation and dysregulate lipid homeostasis, both key features in the pathogenesis of dyslipidemia. Our findings align with previous studies linking lower *Coprococcus* spp. abundance to higher triglyceride levels (22) and atherogenic lipid profiles (23), underscoring the potential role of SCFA-producing taxa in metabolic health (24).

In contrast, *B. caccae* was enriched in the dyslipidemia group. Although this association was identified only in one statistical model (MaAsLin2), it is noteworthy because *B. caccae* has been implicated in pro-inflammatory conditions such as type 2 diabetes (25), gout (26), and CVD (27). This finding may indicate a shift toward a more inflammatory microbial community in dyslipidemia, although this interpretation needs validation in future mechanistic studies. *Bacteroides* species, including *B. caccae*, can metabolize bile acids and produce lipopolysaccharides (LPS), which can activate toll-like receptor pathways and exacerbate systemic inflammation (28). Increased abundance of such taxa may therefore contribute to the inflammatory and metabolic disturbances observed in dyslipidemia. These microbial shifts suggest a potential mechanism by which gut dysbiosis may contribute to dyslipidemia. However, further experimental validation is required to establish direct causality.

Notably, species-level associations were not apparent in unadjusted analyses but emerged after controlling for host covariates such as age, sex, BMI, blood glucose, and blood pressure. This underscores the importance of rigorous covariate adjustment in observational microbiome studies, as host metabolic factors can obscure disease-associated microbial signatures. Without such adjustment, disease-associated microbial signatures may be masked by confounder influences, emphasizing the need for rigorous statistical control in observational microbiome studies (29, 30). Exploratory stratified analyses further suggested that dyslipidemia-associated microbial differences were more consistently observed in individuals with higher metabolic risk profiles (i.e., above-average age, BMI, SBP, or fasting glucose), although statistical power was limited within subgroups. These findings highlight the potential for host-microbiome interactions to differ by metabolic context and underline the need for larger stratified cohorts to separate biological effects from sample size limitations (31).

Lipid-trait-specific analyses indicated that hypertriglyceridemia accounted for much of the microbial signal observed in composite dyslipidemia. Notably, all three taxa depleted in dyslipidemia showed consistent depletion in hypertriglyceridemia, and among them, *B. obeum* overlapped with the 16 TG-negatively associated taxa reported by Lu et al. (32), suggesting cross-cohort consistency for this taxon (32). The TG metabolism is closely linked to hepatic lipogenesis, insulin resistance, and bile acid signaling, all of which are influenced by microbial activity (33). Nevertheless, other lipid traits, including TC and LDL-C, also contributed complementary but weaker signals, suggesting that dyslipidemia-associated microbial alterations reflect the overall burden of lipid dysregulation rather than a single lipid abnormality.

Functional profiling revealed dyslipidemia-associated shifts in microbial pathways related to cell wall biosynthesis, amino acid metabolism, and energy storage (34). In this study, we identified functional alterations associated with dyslipidemia, primarily involving pathways related to amino acid metabolism, cell wall biosynthesis, and energy metabolism. Among the enriched pathways, dTDP-beta-D-fucofuranose biosynthesis (PWY-7312) is involved in the formation of LPS O-antigen (35), which can enhance bacterial immune evasion and trigger host inflammation, a mechanism potentially contributing to atherosclerosis (36). Conversely, L-glutamine biosynthesis (PWY-6549) and glycogen biosynthesis (GLYCOGENSYNTH-PWY) were decreased in dyslipidemia, indicating a diminished microbial capacity for anti-inflammatory regulation (37) and energy homeostasis. Our previous research also linked microbial glycogen pathways with metabolic health, supporting this interpretation (38). Notably, peptidoglycan biosynthesis (PWY-5265 and PWY-6470) was reduced in cases of dyslipidemia. Peptidoglycan fragments have been implicated in metabolic inflammation (39) and insulin resistance (40), and their depletion may reflect structural shifts within the gut microbiota. However, the causal relationship between microbial cell wall remodeling and host lipid metabolism remains unclear and warrants future investigation.

Predicted metabolite analyses using MelonnPan identified a limited number of metabolites associated with dyslipidemia, including reduced pseudouridine and marginal alterations in uracil and N-acetylhistidine. These results should be interpreted cautiously, as they are model-derived predictions and were not validated by targeted metabolomics. Nonetheless, correlations between microbial pathways, taxa, and predicted metabolites suggest coordinated functional shifts within the gut ecosystem.

Although the association between dyslipidemia and the tetracycline resistance gene subtype *tetQ* was observed in this study, its effect size was modest, and statistical significance was attenuated after covariate adjustment and multiple-testing correction. Internal robustness analyses suggested directional stability of the association but did not provide evidence for independent replication. Therefore, the *tetQ* finding should be interpreted as exploratory and hypothesis-generating rather than as indicative of a specific mechanistic link. Although *tetQ* has been consistently detected across human gut metagenomes globally (41), its role in host metabolic health remains unclear. The *tetQ* has been implicated in promoting cytokine responses such as IL-6 and TNF-α and disrupting gut barrier integrity, thereby contributing to systemic inflammation, a known driver of dyslipidemia and cardiovascular risk (42). The presence of *tetQ* on conjugative transposons facilitates its horizontal transfer among gut bacteria (43), especially within *Bacteroides* species (44), potentially driving shifts in microbial community composition. In our study, *Bacteroides stercoris*, which was marginally enriched in dyslipidemia, was positively correlated with *tetQ* abundance, indicating a possible ecological shift favoring pro-inflammatory microbial communities (45). While direct validation using host inflammatory or barrier markers was not feasible in this study, future prospective and experimental studies are needed to explore these pathways in greater depth.

To provide contextual insight into the marginal enrichment of *tetQ*, we explored FFQ-based meat consumption patterns as an exploratory analysis. In South Korea, tetracycline-class antibiotics are widely used in both human medicine and animal production (46), and national surveillance reports indicate a high prevalence of tetracycline resistance among pork-derived bacteria (47). Consistent with this background, higher tetracycline ARG abundance was observed among frequent pork consumers in our cohort. Higher consumption of red and processed meat is associated with an increased risk of dyslipidemia compared to the lowest consumption group in Korea (48). Although dyslipidemia prevalence was higher among frequent beef consumers, no consistent associations were identified between meat consumption and both *tetQ* abundance and dyslipidemia. These findings suggest that dietary patterns may contribute to variation in resistome profiles but do not support a direct link between meat consumption, *tetQ*, and dyslipidemia in this study. Given the exploratory nature of this analysis, incomplete dietary data, and potential residual confounding factors (e.g., antibiotic use in livestock and environmental factors), these observations should be interpreted cautiously and warrant further investigation in studies with comprehensive dietary assessment and targeted resistome profiling.

Despite the strengths of this study, several limitations should be considered. First, its cross-sectional and retrospective design precludes causal inference between gut microbiota and dyslipidemia. Second, the analyses were based on a Korean population, which may limit generalizability to other ethnic groups. Third, although our findings suggest a reduction in SCFA-producing bacteria in dyslipidemia, we could not directly correlate these shifts with host inflammatory or barrier function markers because serum data were unavailable. Future studies integrating microbiome profiling with circulating markers such as IL-6, CRP, LPS, and zonulin are warranted to validate the physiological impact of microbial dysbiosis on host metabolism and immune function. Fourth, associations between microbial pathways and metabolites were inferred using MelonnPan based on gene abundance data. As no targeted metabolomics or metatranscriptomic data were available, our findings reflect functional potential rather than actual metabolic activity or gene expression. Integrative multi-omics studies incorporating transcriptomic and metabolomic measurements will be necessary to validate these functional inferences. Fifth, the exclusion of individuals with diabetes, coronary artery disease, or lipid-lowering therapy enabled a more focused assessment of dyslipidemia but may have resulted in a cohort with relatively mild metabolic phenotypes. This may limit the applicability of our findings to more severe or clinically complex metabolic conditions. Finally, although stratified and interaction analyses suggested that host metabolic status, particularly BMI, may modify microbiome–dyslipidemia associations, statistical power was limited within subgroups. In addition, incomplete dietary information from the FFQ and missing data on certain lifestyle factors, such as smoking and alcohol consumption, may have influenced the precision of covariate-adjusted estimates. Larger studies with more comprehensive and complete metadata will be required to better account for these potential confounders. Importantly, while our study provides associative evidence linking gut microbial composition, functional potential, resistome, and dyslipidemia, mechanistic validation is required to establish causality. Experimental studies using fecal microbiota transplantation (FMT) in germ-free or antibiotic-treated animal models, as well as longitudinal and dietary intervention studies, may help clarify whether the identified microbial and resistome features contribute directly to dyslipidemic phenotypes.

In conclusion, our findings suggest that dyslipidemia is associated with targeted alterations in gut microbial composition and functional potential, particularly involving SCFA-producing taxa and lipid-relevant pathways. These results provide a foundation for future longitudinal and interventional studies aimed at clarifying causal relationships and evaluating the potential of microbiome-informed strategies for metabolic health.

## MATERIALS AND METHODS

### Study participants and exclusion criteria

The current study is part of a Kangbuk Samsung Cohort Study (KSCS) of Korean men and women who underwent comprehensive annual or biennial examinations at the Kangbuk Samsung Hospital Healthcare Screening Center in South Korea (2, 49). Most of the individuals were employees of industrial companies who must undergo a complete medical examination as part of South Korea’s industrial safety and health regulations. A total of 1,710 participants aged between 23–77 years were registered in the study and underwent comprehensive medical examinations between 23 June 2014 and 31 May 2021.

In total, we excluded 326 participants from 1,710 individuals for downstream analysis based on the following exclusion criteria: use of antibiotics within 6 weeks before enrollment (*n* = 61), history of antacids (*n* = 2), history of cancer (*n* = 60), history of diabetes (*n* = 96), history of stroke (*n* = 9), history of heart disease (*n* = 12), history of coronary disease (*n* = 21), medication history for glucose-lowering drugs (*n* = 75), or use of lipid-lowering drugs (*n* = 139) within the past year. We selected the criteria based on established research indicating that certain conditions and medications can significantly alter the gut microbiota composition, thereby potentially confounding the study’s findings. For instance, the use of antibiotics and antacids, as stated in our criteria, has a profound impact on gut microbiota (50). The 6-week exclusion period was chosen based on previous studies showing that gut microbial communities could generally recover to near-baseline composition within this timeframe (51). Excluding individuals with a history of conditions such as diabetes, cancer, or heart disease (52) is crucial. These conditions are often associated with altered gut microbiota. These conditions and treatments can confound the relationship between gut microbiome and dyslipidemia, potentially skewing results or obscuring the true nature of this relationship. Therefore, excluding participants with these conditions or medication histories aids in ensuring that the study more accurately assesses the impact of the gut microbiome on dyslipidemia, free from these confounding influences. We have also used similar criteria in our previous study. In total, 1,384 participants were included in the final analysis.

This study was a retrospective study using fecal and blood samples collected during the KSCS between 23 June 2014 and 31 May 2021. All samples were obtained on the date of participant consent during the routine health screening.

### Data collection and group definition

Data on medical history, medication usage, alcohol use, and sociodemographic characteristics were collected using a self-administered, structured questionnaire. Dietary consumption was assessed using a 103-item self-administered FFQ developed for use in Korea (https://www.nih.go.kr/ko/cmmn/file/fileDown.do?atchFileId=13288349307a4b4a9075d54bb739b267&fileSn=5&bbsId=B0000009) (53). This validated FFQ was designed to measure everyday food consumption during the previous year. The trained hospital staff measured blood pressure and anthropometric parameters during health examinations (54). BMI was calculated by dividing weight (kg) by square of height (m^2^). Blood samples were collected after at least 10 h of fasting. The serum levels of TG and TC were measured with an enzymatic colorimetric assay. Both LDL-C and HDL-C were measured directly with homogeneous enzymatic colorimetric assays. Fecal samples were collected from participants, frozen within five minutes at −20°C on defecation, and stored at −70°C within 24 h of sampling until further processing. We first categorized subjects into two groups, the healthy control or dyslipidemia groups, according to blood lipid measurements in the third report of the National Cholesterol Education Program (55). Dyslipidemia was defined as TC ≥ 200 mg/dL, LDL-C ≥ 130 mg/dL, TG ≥ 150 mg/dL, or low HDL-C (HDL-C ≤ 40 mg/dL for men and ≤ 50 mg/dL for women).

### DNA extraction and shotgun sequencing

The OMNIgene-GUT collection kit (OMR-200, DNA Genotek, Ottawa, Canada) was used to collect stool samples. DNA was extracted from fecal samples within 1 month of storage using standard techniques with the DNeasy PowerSoil Pro Kits (Qiagen, Hilden, Germany) as per the manufacturer’s instructions. The concentration and quality of the extracted DNA were measured using a Qubit fluorometer (Invitrogen, CA, USA), and the DNA was stored at −70°C until further processing. Paired-end sequencing (2 × 150 bp) was carried out on the Illumina NovaSeq 6000 platform (2 × 150 bp paired-end reads) at Macrogen, Korea, generating a total of over 10.3 T bp (an average of 7.4 G bp per sample) and 67.8 G reads. Preprocessing of shotgun metagenomic reads was performed by removing the adapter sequences and low-quality reads with Trimmomatic (ver. 0.39) (56). Trimmomatic was run with parameters SLIDINGWINDOW:4:20 MINLEN:75. For the removal of host DNA sequences, all the metagenomic reads were mapped against the human genome (hg38) using BWA (ver. 0.7.17) (57), and Samtools (ver. 1.15.1) tools were used to obtain FastQ reads of non-human DNA, producing 58.7 G reads with a mean (SD) of 42.4 M (11.4 M) reads per sample for the metagenomic analysis. For taxonomic and functional profiling, we used MetaPhlAn3 (ver. 3.0) (58) and HUMAnN3 (ver. 3.0) (58), respectively. These tools offer species-level resolution and pathway-based functional annotation with high accuracy and have been widely validated in human shotgun metagenomic studies (18). The UniRef90 gene output profile obtained from HUMAnN3 was used as input for the MelonnPan package (ver. 0.99.0) in R to predict metabolites in units of relative abundance following the predict_metabolites.R script (19). Unmapped reads were processed with ShortBRED (ver. 0.9.3) (59) using the Comprehensive Antibiotic Resistance Database (mid-2017) (60) as a reference gene marker to define the composition and abundance of ARGs in each sample. Similarly, the Virulence Factors Database (mid-2017) (61) marker genes were used to quantify VFGs with ShortBRED. Reads per kilobase of transcript per million reads mapped (RPKM) were used to denote the abundance of ARGs and VFGs. Bacterial species, metabolic pathways, gut metabolites, ARG subtypes, and VFGs found in < 10% of the samples (prevalence) were removed from downstream analyses.

### Statistical analysis

All the basic statistical analyses were performed using R (ver. 4.3.0, RStudio, Boston, MA, USA) within the R studio environment (ver. 1.2.5033).

To compare the basic characteristics of all the individuals, we used the moonBook (62) package (ver. 0.3.1) in R. To address potential multicollinearity between covariates in the regression model, we conducted a multicollinearity analysis (e.g., Variance Inflation Factor, VIF) for all the significant covariates identified in Table 1. Based on the result of the high collinearity between SBP and DBP, we selected SBP as the representative covariate due to its stronger association with cardiovascular risk (63). This approach was also applied to other statistically significant variables in Table 1 to ensure model stability and avoid redundancy. Based on the results of the multicollinearity analysis, we ultimately used sex, age, BMI, blood glucose levels, and SBP as covariates in our statistical model. The covariates had VIF values less than 4, indicating no significant collinearity issues. Typically, a VIF value greater than 5 or 10 suggests a problematic level of collinearity, while a VIF value of 2.5 or higher indicates considerable collinearity (64). Lipid-related variables were not included as covariates since they were part of the dyslipidemia definition.

To calculate alpha diversity (Simpson, Shannon, Evenness, and observed) and beta diversity (Bray-Curtis dissimilarity, Jaccard dissimilarity, and Aitchison distance) indices for bacterial taxa and ARGs, we used the vegan package (ver. 2.5.6) (65) in R. A linear regression model was utilized for comparative analysis of alpha diversity between groups. The dependent variables in our models were alpha diversity indices for relative abundance of bacterial taxa and normalized abundance based on RPKM of ARGs, whereas the independent variable was a categorical “group” variable distinguishing between dyslipidemia and healthy individuals. To control for potential confounding factors, we included five covariates (sex, age, BMI, blood glucose levels, and SBP) in our models. To further assess the robustness of associations observed for bacterial alpha diversity, two sensitivity analyses were performed: (i) multivariable linear models incorporating five key covariates as interaction terms and (ii) analyses by individual lipid abnormalities, including hypertriglyceridemia, hypercholesterolemia, elevated LDL-C, and low HDL-C.

For beta diversity indices, to test the significance of differences between groups, we performed a PERMANOVA with 999 random permutations in the adonis2 function of the vegan package (65), adjusting for covariates (age, sex, BMI, SBP, and blood glucose levels). In this analysis, covariates (age, sex, BMI, SBP, and blood glucose levels, in that order) were placed first, and the group variable (case/control) was placed last in the model.

To investigate the significant differences in the abundances of bacterial taxa, we used generalized linear models implemented in the MaAsLin2 (17) package (ver. 1.22.0) in R. The analysis focused on species with relative abundance > 0.1% in 10% of individuals. In the MaAsLin2 models, we estimated the coefficients of log-transformed abundance by comparing the dyslipidemia group to the reference control group after adjusting for sex, age, BMI, blood glucose levels, and SBP. Where zeros cause problems with log transformation incorporated into MaAsLin2, a pseudo-count value, which is half of the minimum feature, was added to all zeros. We utilized the taxa-wide false discovery rate (FDR) with the significance level set to FDR-adjusted *q*-value < 0.05 using Benjamini-Hochberg multiple testing correction. In addition, associations with *q*-values between 0.05 and 0.1 were reported as marginally significant to reduce the risk of overlooking potentially meaningful associations due to false-negative findings.

In addition, we conducted three sensitivity analyses to assess the robustness of our findings for differentially abundant bacterial taxa. First, we applied the ANCOM-BC (66) package (ver. 2.10.1) in R, a method designed to address compositional biases in metagenomic data. While MaAsLin2 is well-suited for multivariable modeling with strong FDR control across diverse microbiome study designs, ANCOM-BC provides bias-corrected estimates of differential abundance using a log-linear framework (17). Log-fold changes were obtained, and *p*-values were calculated using two-sided Z-tests. Second, we conducted stratified subgroup analyses based on key clinical variables, age, BMI, SBP, and blood glucose levels, each of which was also included as a covariate in the main models. For each variable, participants were divided into upper and lower strata based on the mean values of the control group for age (44 years; *n* = 787 vs. 597), BMI (22.9 kg/m^2^; *n* = 873 vs. 511), SBP (106.9 mmHg; *n* = 860 vs. 524), and fasting glucose (92.8 mg/dL; *n* = 789 vs. 595). Within each stratum, we re-estimated the associations for the differentially abundant bacterial species using MaAsLin2 and compared the effect sizes and statistical significance with the unstratified analysis. Third, to evaluate whether specific lipid components predominantly drove the observed associations, we conducted analyses by individual lipid abnormalities, including hypertriglyceridemia, hypercholesterolemia, elevated LDL-C, and low HDL-C.

To identify differentially abundant metabolic pathways and gut metabolites between groups, we compared total “unstratified relative abundance” > 0.0% in 10% of individuals using generalized linear models implemented in MaAsLin2. For the MaAsLin2 models, we estimated the coefficients of log-transformed abundance by comparing the dyslipidemia group to the reference control group after adjusting for sex, age, BMI, blood glucose levels, and SBP. Benjamini-Hochberg FDR correction was applied, with significance defined at *q* < 0.05 and 0.05 ≤ *q* < 0.1 considered marginally significant. In addition, to visualize specific species’ contributions to metabolic pathways, we constructed a “stratified relative abundance”-based bacterial contribution graph for metabolic pathways utilizing the humann_barplot function (58).

For comparative analysis of ARGs and VFGs, total sum scaling (TSS)-normalized relative abundance was used as input for MaAsLin2. Group differences were assessed using linear models adjusting for five covariates. Benjamini-Hochberg FDR correction was used, defining significance *q* < 0.05 and marginal significance at 0.05–0.1. Figures were also generated using TSS-normalized values for consistency with model inputs. To evaluate the stability of results in the absence of an external validation cohort, internal validation was applied specifically to the tetracycline resistance gene *tetQ*, the only ARG subtype showing a marginal association with dyslipidemia. We applied stratified five-fold cross-validation (70/30 train-test splits) and bootstrap resampling (1,000 iterations) using the same covariate-adjusted linear models as in the primary analysis, with the aim of assessing effect direction and effect-size stability rather than formal replication.

To investigate the relationship between meat consumption and tetracycline resistance gene abundance in dyslipidemia, we analyzed the prevalence of dyslipidemia and abundance of tetracycline resistance gene according to the frequency of pork and beef consumption. A total of 889 individuals (566 dyslipidemia cases and 323 controls) who responded to the FFQ were included for this analysis. The frequency cutoffs used for analyzing the association between *tetQ* abundance and FFQ data were determined based on the observed distribution of consumption frequencies in our cohort. For both pork and beef, the highest prevalence was found to be consumption frequency of more than two times per month (Fig. S9). The second highest prevalence was found for pork consumed more than once a week and beef consumed more than once a month. We compared individuals who consumed pork at least 1–2 times per week (*n* = 228) and the others (*n* = 661). In addition, we compared individuals who consumed beef more than once per month (*n* = 730) and those who did not (*f* = 159). Then, we used a linear regression model with adjustment for five covariates to examine the association between pork or beef consumption and the abundance of *tetQ*, identified as the differentially abundant ARG between dyslipidemia cases and controls based on the FDR-corrected analysis. Finally, we compared the rate of pork/beef consumers between dyslipidemia cases and controls, using logistic regression models with covariate adjustment. To control confounding factors, five variables, including sex, age, BMI, blood glucose levels, and SBP, were used as covariates for this analysis.

Spearman’s correlation analysis was conducted to assess the relationship between the tetracycline resistance gene subtype *tetQ* and differentially abundant bacterial species. Similarly, Spearman’s correlations were examined among differentially abundant bacterial species, metabolic pathways, and predicted gut metabolites. In addition, we then correlated all the combinations of total ARGs, total VFGs, ARG types, tetracycline subtype ARGs, VFG types, and the phenotypic characters. For correlation analyses involving multiple features, *P*-values were adjusted for multiple testing using the Benjamini-Hochberg FDR procedure. We used the Hmisc (ver. 5.1.1), corrplot (ver. 0.92), and ggplot2 (ver. 3.4.4) packages in R to make the correlation heatmap.

Finally, a schematic figure summarizing all key findings is presented in Fig. S10.

## Data Availability

The raw sequencing data have been deposited in the NCBI Sequence Read Archive (BioProject accession no. PRJNA1195215). All processed data, including relative abundance tables for bacterial species, pathways, ARGs, and VFGs, are provided as Tables S16 to S21, in which the “Sample_ID” column matches the NCBI Sequence Read Archive sample identifiers and the “Group” column specifies dyslipidemia case–control status. The analysis code used in this study is publicly available at Zenodo (https://doi.org/10.5281/zenodo.18397095).

## SUPPLEMENTAL MATERIAL

Data Set S1 spectrum.00971-25-s0001.xlsx Tables S16 to S21.

Supplemental material spectrum.00971-25-s0002.pdf Fig. S1 to S10; Tables S1 to S15.

ASM does not own the copyrights to Supplemental Material that may be linked to, or accessed through, an article. The authors have granted ASM a non-exclusive, world-wide license to publish the Supplemental Material files. Please contact the corresponding author directly for reuse.
