# Supplementary material for: Gut microbial community structure, metabolic signature, and resistome in dyslipidemia: implications for cardiovascular disease management
Source: Microbiol Spectr. 2026 Mar 4;14(4):e00971-25. doi: 10.1128/spectrum.00971-25 (PMC13055376; doi:10.1128/spectrum.00971-25)
Supplement: Supplemental material — Fig. S1 to S10; Tables S1 to S15. [file spectrum.00971-25-s0002.pdf]

Gut Microbial Community Structure, Metabolic Signature, and Resistome in Dyslipidemia: Implications for Cardiovascular Disease Management

Soomin Lee[1,†], Hyung-Lae Kim[2,†], Shahbaz Raza[1], Eun-Ju Lee[1], Yoosoo Chang[1,3,4], Seungho Ryu[1,3,4], Juhee Cho[1,5,6], Han-Na Kim[1,5,*]

[1] Department of Clinical Research Design and Evaluation, Samsung Advanced Institute for Health Sciences and Technology, Sungkyunkwan University, Seoul, Republic of Korea

[2] Department of Biochemistry, College of Medicine, Ewha Womans University, Seoul, Republic of Korea

[3] Center for Cohort Studies, Total Healthcare Center, Kangbuk Samsung Hospital, Sungkyunkwan University School of Medicine, Seoul, Republic of Korea

[4] Department of Occupational and Environmental Medicine, Kangbuk Samsung Hospital, Sungkyunkwan University School of Medicine, Seoul, Republic of Korea

[5] Center for Clinical Epidemiology, Samsung Medical Center, Sungkyunkwan University, Seoul, Republic of Korea

[6] Departments of Epidemiology and Medicine, and Welch Center for Prevention, Epidemiology and Clinical Research, John Hopkins Medical Institutions, Baltimore, United States

† These authors contributed equally to this work.

*** Correspondence:**
Han-Na Kim
E-mail: hanna147942@gmail.com; Tel: +82-2-3410-2427, Fax: + 82-2-3410-6639

Supplementary Material

Supplementary Figures and Tables

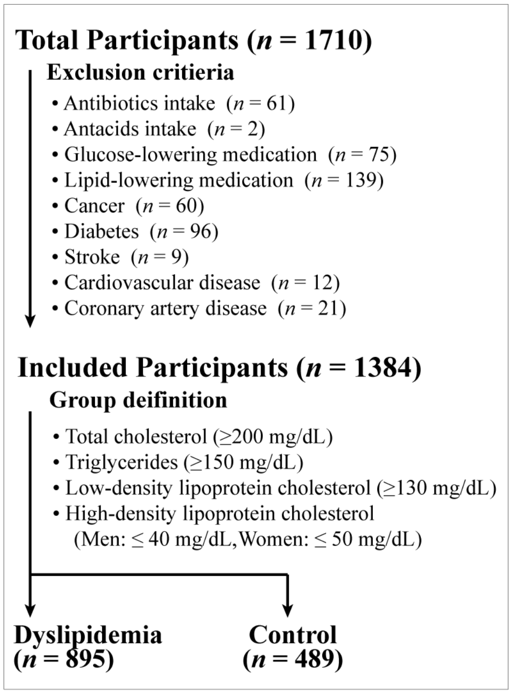

Fig. S1. Exclusion criteria and group definition of this study.

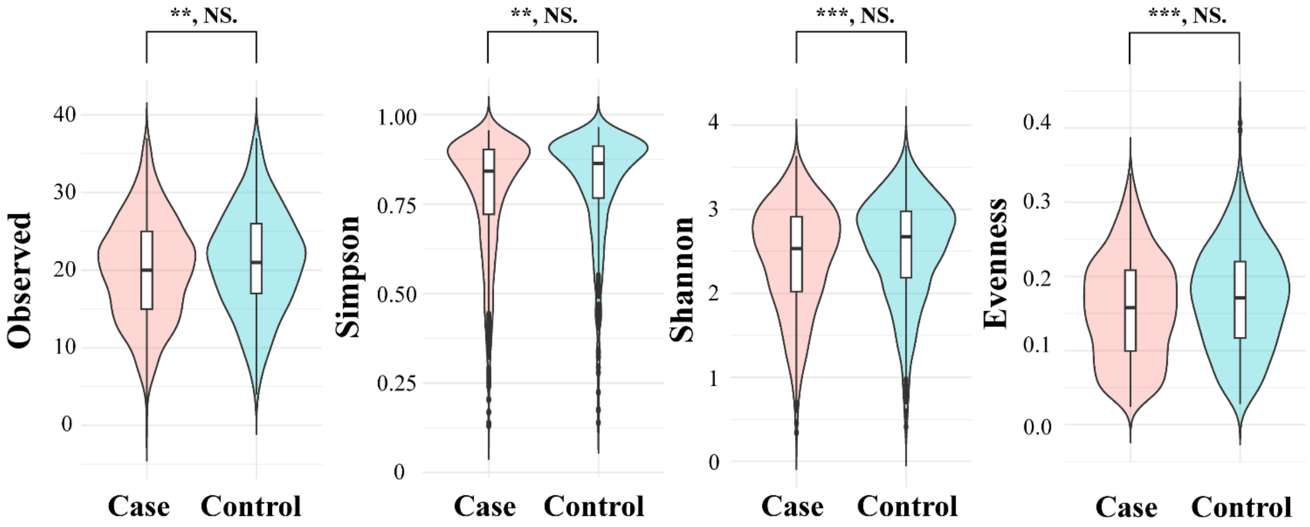

Fig. S2. Alpha diversity analysis for case and control groups based on the relative abundance of bacterial taxa. We applied linear regression model with and without adjusting for covariates, including age, sex, blood glucose levels, body mass index (BMI), and systolic blood pressure (SBP). The *p*-values of the linear regression model were represented for without and with adjusting for covariates. NS., non-significant, *$p < 0.05$, **$p < 0.01$, ***$p < 0.001$.

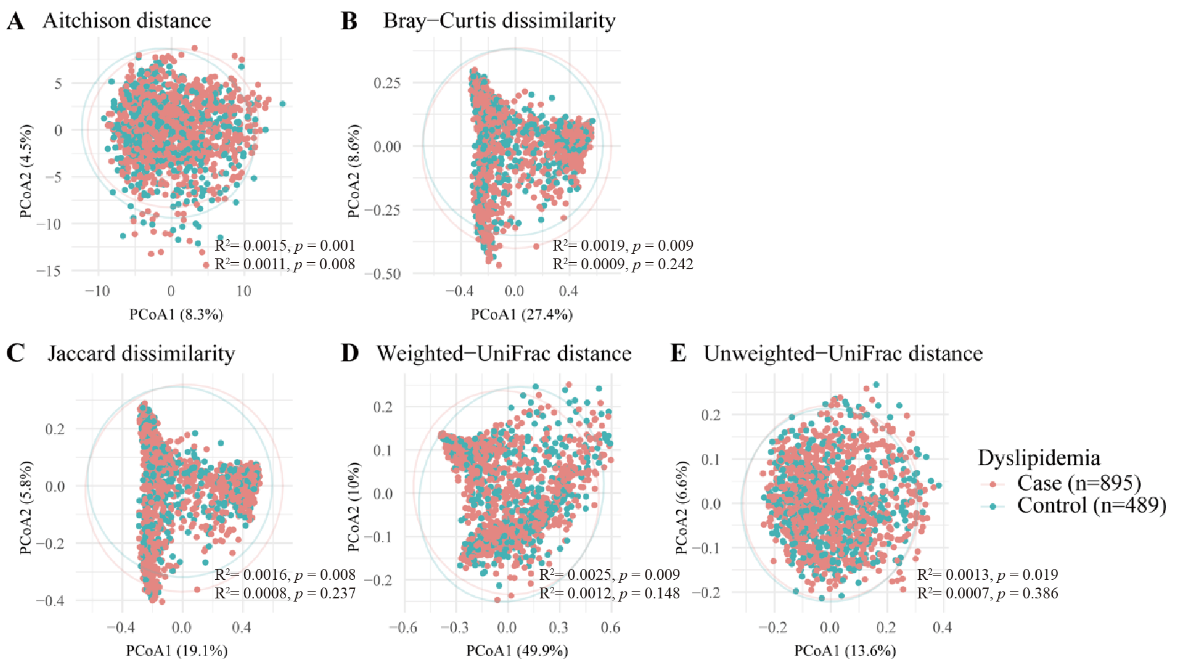

Fig. S3. Principal coordinate analysis based on the Aitchison distance (A), Bray-Curtis dissimilarity (B), Jaccard dissimilarity (C), weighted UniFrac distance (D), and unweighted UniFrac distance (E) of the abundance of bacterial species observed in all the samples. Differences in beta-diversity between groups were evaluated using permutational multivariable analysis of variance (PERMANOVA) with 999 permutations, without and with adjustment for age, sex, body mass index (BMI), and systolic blood pressure (SBP), and blood glucose levels.

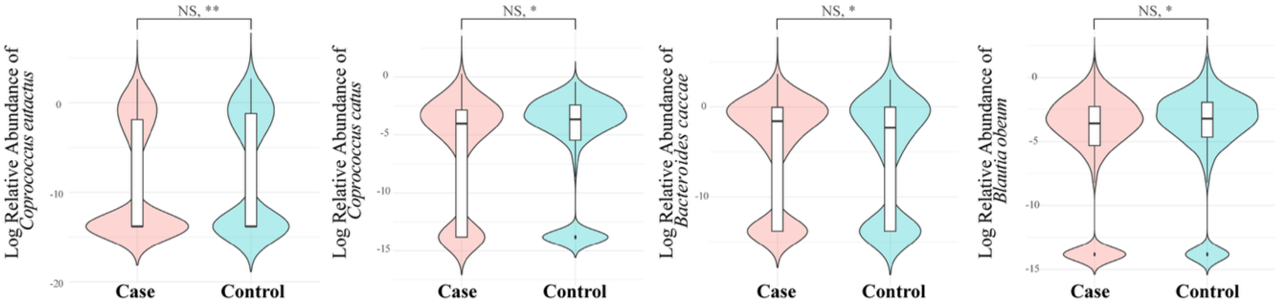

Fig. S4. Violin plot for relative abundance of four significantly differentially abundant bacterial species between case and control groups. The relative abundance data were log-transformed. We applied linear regression model before and after adjusting for five covariates, including age, sex, blood glucose levels, body mass index (BMI), and systolic blood pressure (SBP). NS., non-significant, *$p < 0.05$, **$p < 0.01$, ***$p < 0.001$.

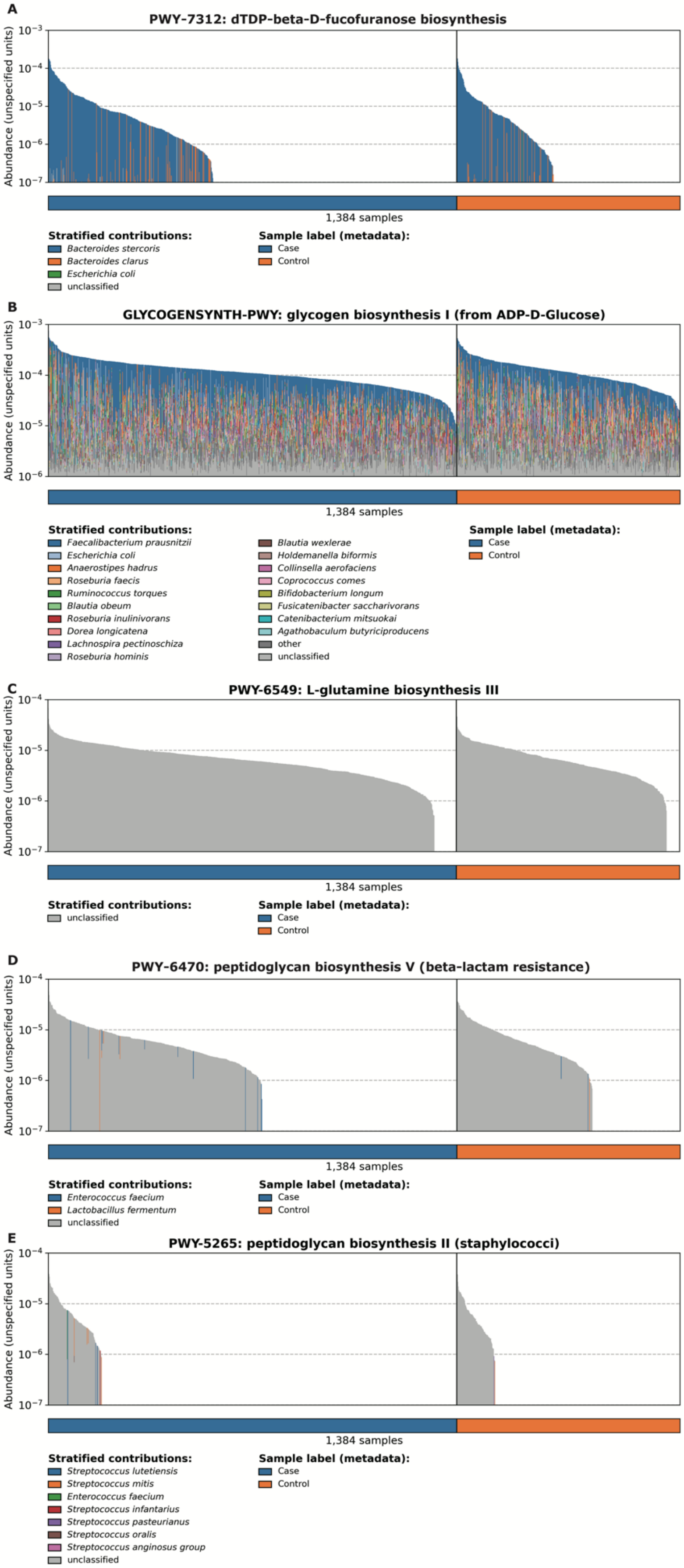
A
PWY-7312: dTDP-beta-D-fucofuranose biosynthesis
Abundance (unspecified units)
10^{-3}
10^{-4}
10^{-5}
10^{-6}
10^{-7}
1,384 samples
Stratified contributions:
Bacteroides stercoris
Bacteroides clarus
Escherichia coli
unclassified
Sample label (metadata):
Case
Control
B
GLYCOGENSYNTH-PWY: glycogen biosynthesis I (from ADP-D-Glucose)
Abundance (unspecified units)
10^{-3}
10^{-4}
10^{-5}
10^{-6}
1,384 samples
Stratified contributions:
Faecalibacterium prausnitzii
Escherichia coli
Anaerostipes hadrus
Roseburia faecis
Ruminococcus torques
Blautia obeum
Roseburia inulinivorans
Dorea longicatena
Lachnospira pectinoschiza
Roseburia hominis
Blautia wexlerae
Holdemanella biformis
Collinsella aerofaciens
Coprococcus comes
Bifidobacterium longum
Fusicatenibacter saccharivorans
Catenibacterium mitsuokai
Agathobaculum butyriciproducens
other
unclassified
Sample label (metadata):
Case
Control
C
PWY-6549: L-glutamine biosynthesis III
Abundance (unspecified units)
10^{-4}
10^{-5}
10^{-6}
10^{-7}
1,384 samples
Stratified contributions:
unclassified
Sample label (metadata):
Case
Control
D
PWY-6470: peptidoglycan biosynthesis V (beta-lactam resistance)
Abundance (unspecified units)
10^{-4}
10^{-5}
10^{-6}
10^{-7}
1,384 samples
Stratified contributions:
Enterococcus faecium
Lactobacillus fermentum
unclassified
Sample label (metadata):
Case
Control
E
PWY-5265: peptidoglycan biosynthesis II (staphylococci)
Abundance (unspecified units)
10^{-4}
10^{-5}
10^{-6}
10^{-7}
1,384 samples
Stratified contributions:
Streptococcus lutetiensis
Streptococcus mitis
Enterococcus faecium
Streptococcus infantarius
Streptococcus pasteurianus
Streptococcus oralis
Streptococcus anginosus group
unclassified
Sample label (metadata):
Case
Control

Fig. S5. Stratified contribution of bacterial species in selected metabolic pathway. dTDP-beta-D-fucofuranose biosynthesis (**A**), glycogen biosynthesis I (from ADP-D-Glucose) (**B**), L-glutamine biosynthesis III (**C**), peptidoglycan biosynthesis V (beta-lactam resistance) (**D**), and peptidoglycan biosynthesis II (staphylococci) (**E**). The figure was sorted by the sum of metadata, and logstack scaling was applied. For glycogen biosynthesis I pathway (**B**), the cutoff point for grouping bacterial species into the 'others' category is an average relative abundance of 2.75×10^{-5} in the dyslipidemia group.

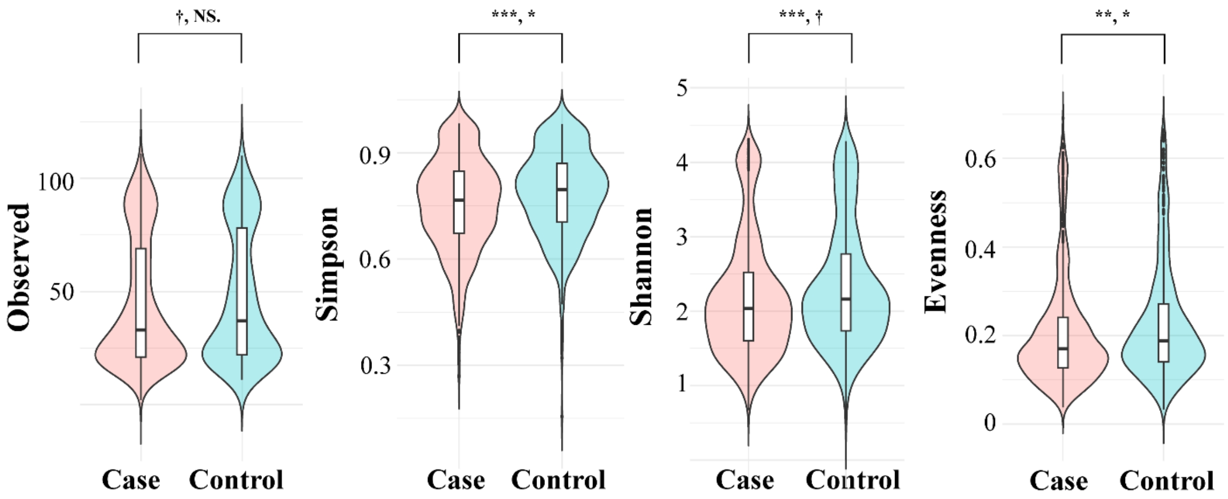

Fig. S6. Alpha diversity analysis for case and control groups based on the abundance of antimicrobial resistance gene (ARG) subtypes. We applied linear regression model with and without adjusting for covariates, including age, sex, blood glucose levels, body mass index (BMI), and systolic blood pressure (SBP). The *p*-values of the linear regression model were represented for without and with adjusting for covariates. NS., non-significant, $^{\dagger}p < 0.1$, $^{*}p < 0.05$, $^{**}p < 0.01$, $^{***}p < 0.001$.

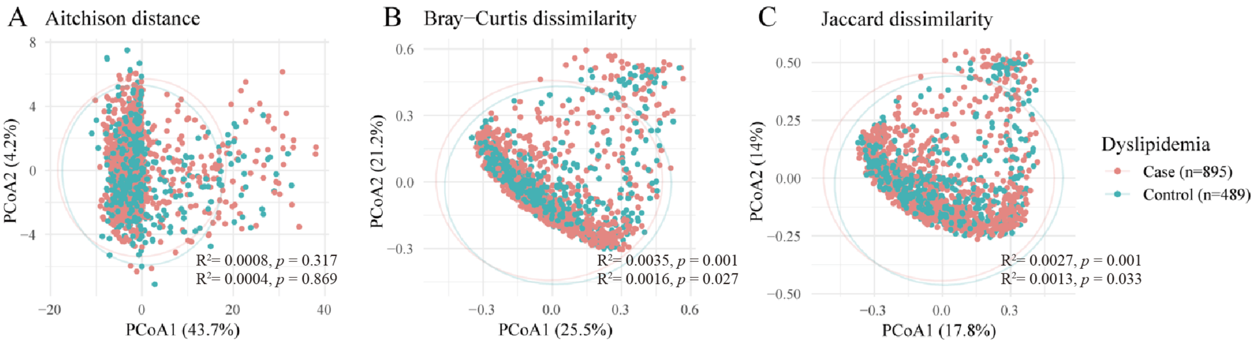

Fig. S7. Principal coordinate analysis based on the Aitchison distance (A), Bray-Curtis dissimilarity (B), and Jaccard dissimilarity (C) of abundance (RPKM) of ARGs in all the samples. Differences in beta-diversity between groups were evaluated using PERMANOVA with 999 permutations, without and with adjustment for age, sex, BMI, SBP, and blood glucose levels.

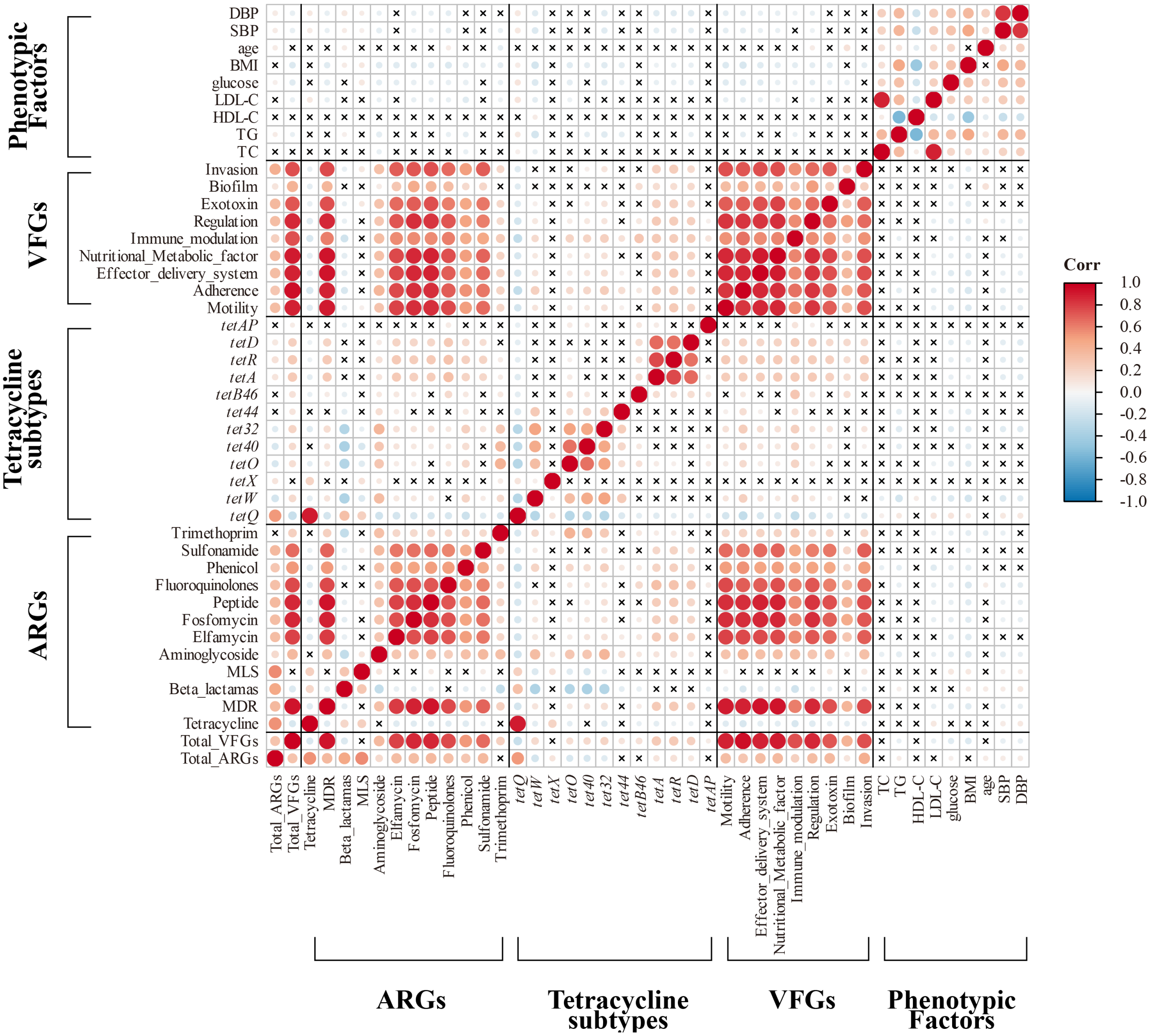

Fig. S8. Correlation analysis of total antimicrobial resistance gene (ARG), total virulence factor gene (VFG), ARG class types, VFG types, and demographic phenotypic characteristics of all participants. The correlation coefficients and *p*-values were calculated using Spearman's correlation test. The *p*-values were adjusted using the Benjamini-Hochberg multiple testing correction. Only significant correlations ($q < 0.05$) are shown in color, while crosses indicate non-significant correlations. The colors represent the Spearman's correlation coefficient ranged from -1.0 to 1.0. The cross mark represents non-significant correlation. Abbreviations. MDR, multidrug-resistance; MLS, macrolide-lincosamide-streptogramin; TC, total cholesterol; TG, triglycerides; LDL-C, low-density lipoprotein cholesterol; HDL-C, high-density lipoprotein cholesterol. $^{*}q < 0.05$, $^{**}q < 0.01$, $^{***}q < 0.001$.

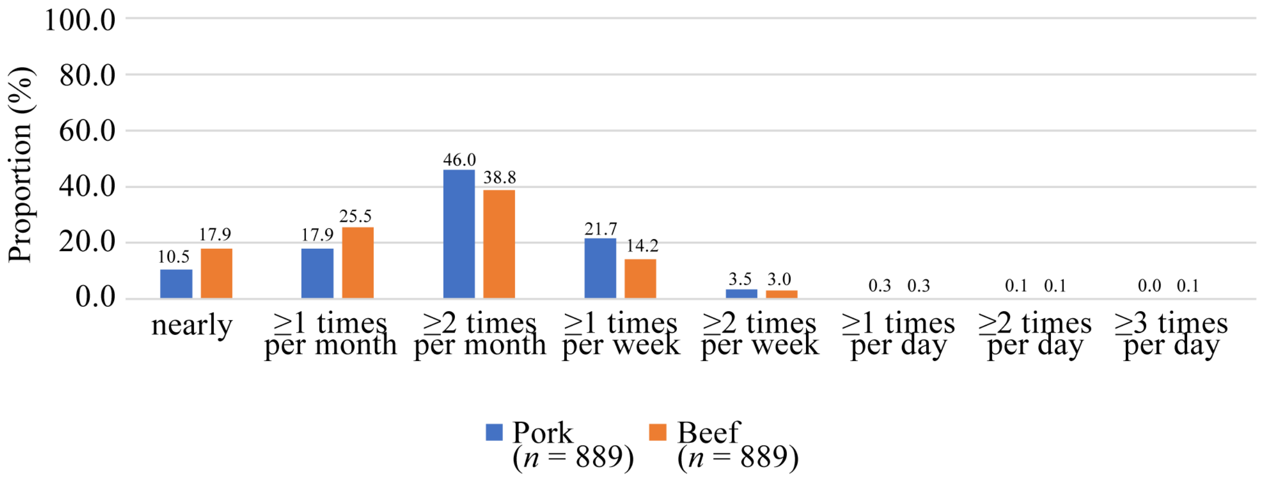

Fig. S9. Beef and pork meat consumption frequency**.**

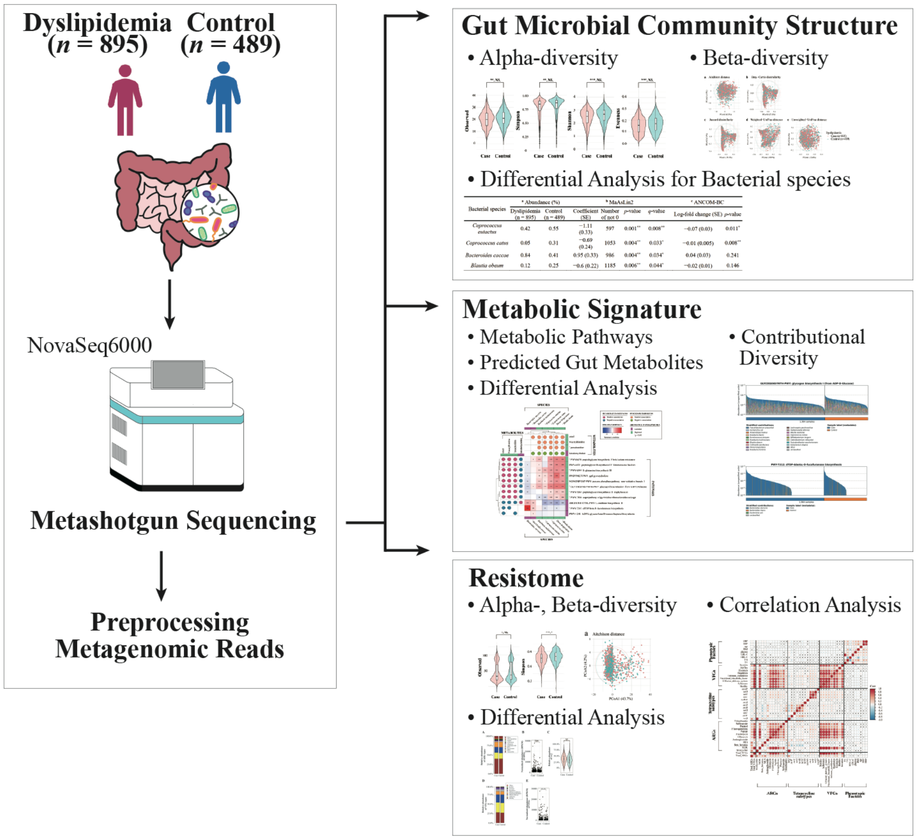

Fig. S10. Study design and analytical workflow. Schematic overview of cohort design, metagenomic sequencing and profiling, downstream statistical analysis.

Table S1. Multivariable linear regression analyses of alpha diversity indices according to dyslipidemia status before and after covariate adjustment, with interaction terms.

		Observed richness		Shannon entropy		Simpson index		Pielou's evenness	
	Variable	Coefficient	*p*-value	Coefficient	*p*-value	Coefficient	*p*-value	Coefficient	*p*-value
Model without adjustment	Group	−0.97	0.008	−0.12	<0.001	−0.03	0.002	−0.01	<0.001
Model without interaction term	Age	−5.00E−03	0.825	−3.04E−04	0.88	−9.07E−04	0.073	−4.57E−04	0.044*
	Sex	−1.37	0.001**	−0.14	<0.001***	−3.94E−02	<0.001***	−1.68E−02	<0.001***
	BMI	−0.21	0.001**	−0.02	<0.001***	−5.46E−03	<0.001***	−1.80E−03	0.008**
	SBP	−0.01	0.37	−1.00E−03	0.655	4.01E−05	0.917	−5.14E−05	0.766
	Blood glucose levels	5.00E−03	0.733	4.47E−04	0.725	2.65E−04	0.406	−8.82E−05	0.539
	Group	−0.32	0.399	−0.05	0.137	−1.12E−02	0.218	−5.78E−03	0.158
Model with Age*Group with interaction term	Age	0.02	0.644	3.00E−03	0.427	−4.69E−04	0.571	1.02E−04	0.784
	Sex	−1.4	0.001	−0.15	<0.001***	−3.99E−02	<0.001***	−1.75E−02	<0.001***
	BMI	−0.21	0.001	−0.02	<0.001***	−5.55E−03	<0.001***	−1.92E−03	0.005**
	SBP	−0.01	0.373	−1.00E−03	0.661	4.20E−05	0.913	−4.90E−05	0.776
	Blood glucose levels	0.01	0.707	1.00E−03	0.687	2.75E−04	0.39	−7.56E−05	0.598
	Group	1.17	0.562	0.16	0.415	2.02E−02	0.674	3.44E−02	0.11
	Age*Group interaction	−0.03	0.451	−5.00E−03	0.264	−6.95E−04	0.505	−8.87E−04	0.058
Model with Sex*Group with interaction term	Age	−0.01	0.804	−2.66E−04	0.896	−8.90E−04	0.081	−4.65E−04	0.042*
	Sex	−1.26	0.045*	−0.15	0.011*	−4.25E−02	0.005**	−1.54E−02	0.022*
	BMI	−0.21	0.001**	−0.02	<0.001***	−5.46E−03	<0.001***	−1.81E−03	0.008**
	SBP	−0.01	0.37	−1.00E−03	0.656	4.04E−05	0.916	−5.15E−05	0.765
	Blood glucose levels	5.00E−03	0.722	4.34E−04	0.733	2.59E−04	0.418	−8.56E−05	0.552
	Group	−0.21	0.742	−0.061	0.301	−1.46E−02	0.327	−4.29E−03	0.52
	Sex*Group interaction	−0.18	0.812	0.01	0.876	5.20E−03	0.776	−2.32E−03	0.778
Model with BMI*Group with interaction term	Age	−0.01	0.486	−1.00E−03	0.632	−1.03E−03	0.044*	−5.35E−04	0.019*
	Sex	−1.5	<0.001***	−0.15	<0.001***	−4.09E−02	<0.001***	−1.78E−02	<0.001***
	BMI	0.11	0.311	−2.00E−03	0.877	−1.79E−03	0.485	5.96E−04	0.603
	SBP	−0.01	0.374	−1.00E−03	0.66	4.22E−05	0.912	−5.01E−05	0.771
	Blood glucose levels	0.01	0.679	1.00E−03	0.688	2.76E−04	0.387	−8.10E−05	0.571
	Group	10.19	<0.001***	0.64	0.021*	1.12E−01	0.107	7.44E−02	0.017*
	BMI*Group interaction	−0.45	<0.001***	−0.03	0.012*	−5.22E−03	0.074	−3.41E−03	0.009**

Coefficients and *p*-values were estimated using the linear regression models adjusted for age, sex, body mass index (BMI), systolic blood pressure (SBP), and blood glucose level. **p* < 0.05, ***p* < 0.01, ****p* < 0.001.

Table S2. Alpha diversity differences according to the individual lipid abnormalities

	Variable	Observed richness		Shannon entropy		Simpson index		Pielou's evenness	
		Coefficient	*p*-value	Coefficient	*p*-value	Coefficient	*p*-value	Coefficient	*p*-value
Dyslipidemia case (*n* = 895) vs. control (*n* = 489, ref)	Without adjustment	−0.97	0.008**	−0.12	<0.001***	−0.03	0.002***	−0.01	<0.001***
	With covariates adjustment	−0.32	0.399	−0.05	0.137	−1.12E−02	0.218	−5.78E−03	0.158
Dyslipidemia case without High TG (*n* = 240) vs. control without High TG (*n* = 489, ref)	Without adjustment	−1.40	0.007**	−0.16	0.001**	−0.04	0.003**	−0.01	0.011*
	With covariates adjustment	−0.81	0.153	−0.09	0.090	−0.02	0.150	−6.53−03	0.280
High TG (*n* = 335) vs. Low TG (*n* = 1049, ref)	Without adjustment	−2.20	<0.001***	−0.21	<0.001***	−0.05	<0.001***	−0.02	<0.001***
	With covariates adjustment	−1.40	0.001**	−0.13	0.002**	−0.03	0.003**	−9.02E−03	0.054
High TC (*n* = 655) vs. Low TC (*n* = 729, ref)	Without adjustment	−0.36	0.311	−0.05	0.124	−0.01	0.171	−0.01	0.030*
	With covariates adjustment	−0.07	0.836	−0.024	0.465	−4.40E−03	0.601	−4.43E−03	0.241
High LDL-C (*n* = 596) vs. Low LDL-C (*n* = 788, ref)	Without adjustment	−0.30	0.405	−0.04	0.2	−0.01	0.203	−0.01	0.178
	With covariates adjustment	0.33	0.358	0.02	0.571	5.28E−03	0.543	2.51E−03	0.520
Low HDL-C (*n* = 216) vs. High HDL-C (*n* = 1168, ref)	Without adjustment	−0.12	0.813	−0.02	0.636	−0.01	0.474	−0.001	0.826
	With covariates adjustment	0.26	0.600	0.018	0.708	1.92E−03	0.871	2.81−03	0.598

Coefficients and *p*-values were estimated using the linear regression models with and without adjustment for age, sex, body mass index (BMI), systolic blood pressure (SBP), and blood glucose level. Abbreviations. TG, triglycerides; TC, total cholesterol; LDL-C, low-density lipoprotein cholesterol; HDL-C, high-density lipoprotein cholesterol; ref, reference group. *$p < 0.05$, **$p < 0.01$, ***$p < 0.001$

Table S3. Average relative abundance and coefficients of bacterial species with adjusting for covariates.

Bacterial species	Relative abundance (%)[a]		MaAsLin2[b]				ANCOM-BC[c]	
	Case	Control	Coefficient	Number of not 0	*p*-value	*q*-value	Log-fold change	*p*-value
Coprococcus eutactus	0.420	0.549	−1.111	597	<0.001	0.008 *	−0.071	0.011
Coprococcus catus	0.050	0.062	−0.686	1053	0.004	0.033 *	−0.012	0.008
Bacteroides caccae	0.840	0.887	0.952	986	0.004	0.034 *	0.037	0.241
Blautia obeum	0.116	0.149	−0.603	1185	0.006	0.044 *	−0.016	0.146
Bacteroides stercoris	2.023	1.853	0.978	861	0.012	0.080	0.108	0.025
Roseburia inulinivorans	0.712	0.750	−0.566	1273	0.015	0.096	−0.051	0.047
Dorea longicatena	0.489	0.647	−0.418	1292	0.016	0.098	−0.071	0.002

Adjusted for age, sex, body mass index (BMI), blood glucose levels, and systolic blood pressure (SBP). [a] Average relative abundance (%) of bacterial species in dyslipidemia case and control. [b] The coefficient and *p*-values were calculated with adjusting for covariates using the generalized linear model implemented in Multivariate Association with Linear Models (MaAsLin2). The *q*-values were calculated using the Benjamini-Hochberg method for multiple testing correction. [c] Log-fold changes were obtained from the Analysis of Compositions of Microbiomes with Bias Correction program (ANCOM-BC) log-linear model. The *p*-values were calculated from two-sided Z-test. * *q*-value < 0.05

Table S4. Stratified analysis of differentially abundant bacterial species in dyslipidemia across age, body mass index (BMI), systolic blood pressure (SBP), and fasting glucose level subgroups.

		Differentially abundant bacterial species in dyslipidemia cases and controls			
	Statistics	*Coprococcus eutactus*	*Coprococcus catus*	*Bacteroides caccae*	*Blautia obeum*
All participants (*n* = 1384)	Coefficient	−1.11	−0.69	0.95	−0.6
	SE	0.33	0.24	0.33	0.22
	N	1384	1384	1384	1384
	N.not.0	597	1053	986	1185
	p-value	6.490E−04***	0.004**	0.004**	0.006**
Age ≥ 44 years subgroup (*n* = 787)	Coefficient	−1.45	−0.73	0.93	−0.59
	SE	0.44	0.3	0.4	0.29
	N	787	787	787	787
	N.not.0	388	625	572	676
	p-value	0.001**	0.016*	0.019*	0.041*
Age < 44 years subgroup (*n* = 597)	Coefficient	−0.52	−0.61	0.82	−0.7
	SE	0.43	0.38	0.54	0.34
	N	597	597	597	597
	N.not.0	209	428	414	509
	p-value	0.229	0.11	0.125	0.040*
BMI ≥ 22.9 subgroup (*n* = 873)	Coefficient	−1.16	−0.82	1.21	−0.88
	SE	0.36	0.32	0.42	0.29
	N	873	873	873	873
	N.not.0	376	651	635	746
	p-value	0.001**	0.009**	0.004**	0.002**
BMI < 22.9 subgroup (*n* = 511)	Coefficient	−0.78	−0.59	0.4	−0.3
	SE	0.51	0.36	0.47	0.34
	N	511	511	511	511
	N.not.0	221	402	351	439
	p-value	0.125	0.1	0.393	0.378
SBP ≥ 106.9 mmHg subgroup (*n* = 860)	Coefficient	−1.55	−1.22	1.32	−0.8
	SE	0.43	0.32	0.43	0.29
	N	860	860	860	860
	N.not.0	372	636	610	729
	p-value	3.281E−04***	1.488E−04***	0.002**	0.006**
SBP < 106.9 mmHg subgroup (*n* = 524)	Coefficient	−0.48	0.02	0.32	−0.32
	SE	0.45	0.35	0.49	0.34
	N	524	524	524	524
	N.not.0	225	417	376	456
	p-value	0.285	0.956	0.512	0.346
Blood glucose levels ≥ 92.8 mg/dL subgroup (*n* = 789)	Coefficient	−1.81	−1.04	1.42	−0.72
	SE	0.43	0.33	0.45	0.3
	N	789	789	789	789
	N.not.0	323	585	567	668
	p-value	3.215E−05***	0.002**	0.002**	0.016*
Blood glucose levels < 92.8 mg/dL subgroup (*n* = 595)	Coefficient	−0.29	−0.29	0.29	−0.49
	SE	0.42	0.34	0.45	0.32
	N	595	595	595	595
	N.not.0	274	468	419	517
	p-value	0.487	0.383	0.527	0.128

Abbreviations. SE, standard error; N, total number of participants; N.not.0, number of participants with non-zero counts; BMI, body mass index; SBP, systolic blood pressure. *$p < 0.05$, **$p < 0.01$, ***$p < 0.001$

Table S5. Differential abundance of gut bacterial species across individual lipid abnormalities with adjusting for covariates.

	Differentially abundant bacterial species in dyslipidemia cases and controls				
	Statistics	*Coprococcus eutactus*	*Coprococcus catus*	*Bacteroides caccae*	*Blautia obeum*
Dyslipidemia case (*n* = 895) vs. control (*n* = 489, ref)	Coefficient	−1.11	−0.69	0.95	−0.6
	SE	0.33	0.24	0.33	0.22
	N	1384	1384	1384	1384
	N.not.0	597	1053	986	1185
	p-value	<0.001***	0.004**	0.004**	0.006**
Dyslipidemia case without High TG (*n* = 240) vs. control without High TG (*n* = 489, ref)	Coefficient	−1.61	−0.92	1.22	-0.87
	SE	0.43	0.35	0.49	0.31
	N	729	729	729	729
	N.not.0	322	562	509	633
	p-value	<0.001***	0.008**	0.013*	0.006*
High TG (*n* = 335) vs. Low TG (*n* = 1049, ref)	Coefficient	−0.83	−1.08	0.67	−0.91
	SE	0.37	0.27	0.38	0.25
	N	1384	1384	1384	1384
	N.not.0	597	1053	986	1185
	p-value	0.027*	<0.001***	0.080	<0.001***
High TC (*n* = 655) vs Low TC (*n* = 729, ref)	Coefficient	−0.41	−0.35	0.45	−0.32
	SE	0.3	0.22	0.31	0.2
	N	1384	1384	1384	1384
	N.not.0	597	1053	986	1185
	p-value	0.174	0.116	0.146	0.118
High LDL-C (*n* = 596) vs. Low LDL-C (*n* = 788, ref)	Coefficient	−0.72	−0.33	0.5	−0.3
	SE	0.31	0.23	0.32	0.21
	N	1384	1384	1384	1384
	N.not.0	597	1053	986	1185
	p-value	0.020*	0.140	0.111	0.155
Low HDL-C (*n* = 216) vs. High HDL-C (*n* = 1168, ref)	Coefficient	−0.15	-0.32	0.8	0.14
	SE	0.42	0.31	0.43	0.28
	N	1384	1384	1384	1384
	N.not.0	597	1053	986	1185
	p-value	0.722	0.302	0.066	0.613

Abbreviations. SE, standard error; N, total number of participants; N.not.0, number of participants with non-zero counts; BMI, body mass index; SBP, systolic blood pressure; TG, triglycerides; TC, total cholesterol; LDL-C, low-density lipoprotein cholesterol; HDL-C, high-density lipoprotein cholesterol; ref, reference group. $^{*}p < 0.05$, $^{**}p < 0.01$, $^{***}p < 0.001$

Table S6. Average relative abundance and coefficients of metabolic pathways without adjusting for covariates.

Pathways	Relative abundance (%)[a]		MaAsLin2[b]			
	Case	Control	Coefficient	Number of not 0	*p*-value	*q*-value
PWY-7196: superpathway of pyrimidine ribonucleosides salvage	9.015E−05	1.315E−04	−0.556	575	<0.001	0.006
CENTFERM-PWY: pyruvate fermentation to butanoate	5.016E−04	5.696E−04	−0.278	1357	<0.001	0.017
PWY-6590: superpathway of Clostridium acetobutylicum acidogenic fermentation	6.189E−04	7.013E−04	−0.273	1357	<0.001	0.017
PWY-4041: gamma−glutamyl cycle	1.033E−03	1.172E−03	−0.277	1377	<0.001	0.022
SER-GLYSYN-PWY: superpathway of L-serine and glycine biosynthesis I	5.071E−03	5.433E−03	−0.119	1384	<0.001	0.022
PPGPPMET-PWY: ppGpp metabolism	4.137E−04	4.728E−04	−0.296	1370	<0.001	0.025
PWY-6470: peptidoglycan biosynthesis V beta-lactam resistance)	9.569E−04	1.111E−03	−0.387	1318	<0.001	0.025
PWY-7456: beta-(1,4)-mannan degradation	1.623E−03	1.779E−03	−0.169	1382	<0.001	0.025
GLYCOGENSYNTH-PWY: glycogen biosynthesis I (from ADP-D-Glucose)	4.609E−03	4.968E−03	−0.120	1384	<0.001	0.032
DAPLYSINESYN-PWY: L-lysine biosynthesis I	1.523E−03	1.712E−03	−0.239	1379	<0.001	0.037
P164-PWY: purine nucleobases degradation I (anaerobic)	7.908E−04	8.706E−04	−0.191	1382	0.001	0.040
PWY-6471: peptidoglycan biosynthesis IV (Enterococcus faecium)	1.326E−03	1.544E−03	−0.309	1323	0.001	0.040
PWY-6549: L-glutamine biosynthesis III	8.560E−04	9.712E−04	−0.200	1380	0.001	0.040
PWY66-399: gluconeogenesis III	1.242E−03	1.398E−03	−0.293	1340	0.002	0.041

Prior to adjusting for covariates, namely age, sex, body mass index (BMI), blood glucose levels, and systolic blood pressure (SBP). [a] Average relative abundance (%) of bacterial species in dyslipidemia case and control. [b] The coefficient and *p*-values were calculated with adjusting for covariates using the generalized linear model implemented in MaAsLin2. The *q*-values were calculated using the Benjamini-Hochberg method for multiple testing correction. Metabolites were sorted based on highest to lowest co-efficient values obtained from MaAsLin2.

Table S7. Average relative abundance and coefficients of metabolic pathways with adjusting for covariates.

Pathways	Relative abundance (%)[a]		MaAsLin2[b]			
	Case	Control	Coefficient	Number of not 0	*p*-value	*q*-value
PWY-6470: peptidoglycan biosynthesis V (beta-lactam resistance)	0.096	0.111	−0.350	1318	0.003	0.020*
PWY-7312: dTDP-beta-D-fucofuranose biosynthesis	0.030	0.028	0.547	789	0.004	0.026*
PWY-7196: superpathway of pyrimidine ribonucleosides salvage	0.009	0.013	−0.378	575	0.005	0.029*
PWY-6549: L-glutamine biosynthesis III	0.086	0.097	−0.181	1380	0.006	0.033*
GLYCOGENSYNTH-PWY: glycogen biosynthesis I (from ADP-D-Glucose)	0.461	0.497	−0.099	1384	0.008	0.044*
PWY-5265: peptidoglycan biosynthesis II (staphylococci)	0.009	0.012	−0.347	422	0.009	0.045*
NONOXIPENT-PWY: pentose phosphate pathway (non-oxidative branch) I	0.405	0.433	−0.096	1384	0.011	0.054
PWY-6471: peptidoglycan biosynthesis IV (Enterococcus faecium)	0.133	0.154	−0.256	1323	0.012	0.058
PPGPPMET-PWY: ppGpp metabolism	0.041	0.047	−0.213	1370	0.017	0.077
PWY-1241: ADP-L-glycero-beta-D-manno-heptose biosynthesis	0.071	0.067	0.149	1381	0.018	0.079
ARGININE-SYN4-PWY: L-ornithine biosynthesis II	0.424	0.406	0.192	1380	0.019	0.083

After adjusting for covariates, namely age, sex, body mass index (BMI), blood glucose levels, and systolic blood pressure (SBP). [a] Average relative abundance (%) of bacterial species in dyslipidemia case and control. [b] The coefficient and *p*-values were calculated with adjusting for covariates using the generalized linear model implemented in MaAsLin2. The *q*-values were calculated using the Benjamini-Hochberg method for multiple testing correction. Metabolites were sorted based on highest to lowest co-efficient values obtained from MaAsLin2. * *q*-value < 0.05

Table S8. Average relative abundance of top 50 bacterial species contributing to the glycogen biosynthesis I pathway (GLYCOGENSYNTH-PWY)

Bacterial Species	Average relative abundance %			Sample number of not 0
	Case	Control	Total	
Faecalibacterium prausnitzii	8.12E−04	7.62E−04	7.94E−04	1338
unclassified	3.60E−04	4.06E−04	3.76E−04	1366
Escherichia coli	1.41E−04	1.37E−04	1.40E−04	549
Anaerostipes hadrus	1.12E−04	1.24E−04	1.16E−04	1029
Roseburia faecis	9.34E−05	1.14E−04	1.01E−04	900
Ruminococcus torques	8.07E−05	8.78E−05	8.32E−05	1038
Roseburia inulinivorans	6.58E−05	6.91E−05	6.70E−05	965
Blautia obeum	6.1E−05	7.42E−05	6.57E−05	916
Collinsella aerofaciens	5.59E−05	7.17E−05	6.14E−05	612
Dorea longicatena	5.45E−05	6.90E−05	5.96E−05	1013
Lachnospira pectinoschiza	5.24E−05	4.63E−05	5.03E−05	572
Roseburia hominis	5.23E−05	6.33E−05	5.62E−05	767
Blautia wexlerae	4.8E−05	4.92E−05	4.84E−05	1001
Coprococcus comes	4.41E−05	5.53E−05	4.81E−05	894
Holdemanella biformis	4.4E−05	5.97E−05	4.96E−05	458
Bifidobacterium longum	4.03E−05	5.37E−05	4.50E−05	645
Catenibacterium mitsuokai	3.88E−05	4.12E−05	3.97E−05	351
Fusicatenibacter saccharivorans	3.69E−05	4.18E−05	3.86E−05	745
Eubacterium eligens	2.76E−05	4.09E−05	3.23E−05	395
Agathobaculum butyriciproducens	2.74E−05	3.07E−05	2.86E−05	750
Eubacterium hallii	2.64E−05	3.16E−05	2.82E−05	558
Eubacterium siraeum	2.44E−05	3.47E−05	2.81E−05	261
Roseburia intestinalis	2.42E−05	2.18E−05	2.33E−05	431
Klebsiella pneumoniae	2.13E−05	2.98E−05	2.43E−05	254
Megasphaera elsdenii	1.82E−05	1.38E−05	1.67E−05	201
Flavonifractor plautii	1.78E−05	2.22E−05	1.94E−05	498
Ruminococcus lactaris	1.73E−05	1.85E−05	1.77E−05	415
Mitsuokella jalaludinii	1.62E−05	1.84E−05	1.70E−05	179
Lactobacillus rogosae	1.47E−05	1.10E−05	1.34E−05	209
Dorea formicigenerans	1.45E−05	1.76E−05	1.56E−05	598
Haemophilus parainfluenzae	1.43E−05	1.63E−05	1.50E−05	331
Coprococcus eutactus	1.22E−05	1.72E−05	1.39E−05	166
Fusobacterium mortiferum	1.16E−05	8.14E−06	1.04E−05	61
Bacteroides thetaiotaomicron	1.03E−05	7.49E−06	9.28E−06	248
Lactobacillus ruminis	1.02E−05	9.3E−06	9.88E−06	152
Enterobacter cloacae complex	8.16E−06	3.96E−06	6.68E−06	99
Bacteroides galacturonicus	6.84E−06	9.48E−06	7.78E−06	105
Bifidobacterium bifidum	6.06E−06	6.33E−06	6.16E−06	174

Streptococcus salivarius	5.18E−06	5.6E−06	5.33E−06	176
Aeromonas caviae	4.69E−06	0	3.03E−06	2
Eubacterium ramulus	4.62E−06	5.38E−06	4.89E−06	193
Clostridium sp CAG 299	4.38E−06	2.64E−06	3.76E−06	120
Clostridium clostridioforme	4.09E−06	6.89E−07	2.89E−06	35
Klebsiella variicola	2.84E−06	2.87E−06	2.85E−06	133
Roseburia sp CAG 471	2.16E−06	2.48E−06	2.27E−06	110
Mitsuokella multacida	2.09E−06	2.28E−06	2.16E−06	26
Citrobacter freundii	1.99E−06	2.82E−06	2.28E−06	105
Clostridium bolteae	1.94E−06	8.75E−07	1.57E−06	55
Coprococcus catus	1.78E−06	2.34E−06	1.98E−06	147
Clostridium disporicum	1.68E−06	5.27E−06	2.95E−06	20
Others	2.36E−07	2.95E−07	2.57E−07	

Table S9. Average of the relative abundance and coefficients of gut metabolite predicted from UniRef90 genes without adjusting for covariates.

Metabolites	Relative abundance (%)[a]		MaAsLin2[b]			
	Case	Control	Coefficient	Number of not 0	*p*-value	*q*-value
Undecanedionate	5.619E−05	6.194E−05	−0.172	1384	<0.001	0.008
Ketodeoxycholate	5.893E−04	5.424E−04	0.138	1384	<0.001	0.008
X7 methylguanine	3.008E−04	2.939E−04	0.034	1384	<0.001	0.008
X3 methylxanthine	2.927E−05	2.858E−05	0.035	1384	<0.001	0.008
Lithocholic acid	9.426E−04	1.028E−03	−0.138	1384	0.001	0.008
Deoxyinosine	3.318E−05	3.418E−05	−0.043	1384	0.001	0.008
ADMA SDMA.	1.113E−04	1.074E−04	0.049	1384	0.001	0.010
Deoxycholic acid	6.735E−03	7.105E−03	−0.079	1384	0.002	0.010
C16.0 ceramide-d18.1.	1.114E−04	1.095E−04	0.0245	1384	0.001	0.010
Thymine	1.634E−04	1.707E−04	−0.062	1384	0.001	0.010
N−acetylputrescine	2.582E−04	2.692E−04	−0.061	1384	0.002	0.010
Threosphingosine	1.868E−04	1.824E−04	0.034	1384	0.002	0.010
Diacetylspermine	9.674E−05	9.330E−05	0.053	1384	0.002	0.010
C20.4 carnitine	1.141E−04	1.177E−04	−0.045	1384	0.001	0.010
Pseudouridine	4.750E−05	4.838E−05	−0.027	1384	0.002	0.011
Chenodeoxycholate deoxycholate.	3.221E−03	3.355E−03	−0.057	1384	0.002	0.011
Cholate	2.498E−03	2.309E−03	0.130	1384	0.003	0.012
Hypoxanthine	6.007E−04	6.030E−04	−0.006	1384	0.003	0.014
Bilirubin	3.478E−04	3.434E−04	0.019	1384	0.004	0.018
X2 hydroxymyristic acid	3.487E−05	3.622E−05	−0.056	1384	0.005	0.019
Urobilin	6.576E−04	7.218E−04	−0.168	1384	0.005	0.019
C18.1.CE	1.063E−04	1.046E−04	0.023	1384	0.006	0.019
N-acetylglutamic acid	6.081E−05	6.324E−05	−0.060	1384	0.006	0.019
C2 carnitine	1.027E−04	9.296E−05	0.143	1384	0.006	0.019
Xanthine	1.445E−03	1.428E−03	0.019	1384	0.006	0.020
C16 carnitine	2.373E−05	2.325E−05	0.030	1384	0.007	0.021
Uracil	5.853E−04	5.959E−04	−0.025	1384	0.008	0.023
Chenodeoxycholate	3.822E−04	3.577E−04	0.105	1384	0.008	0.023
Nicotinate	1.808E−04	1.836E−04	−0.022	1384	0.008	0.023
Sebacate	1.142E−04	1.177E−04	−0.046	1384	0.009	0.024
Cholestenone	9.745E−04	9.965E−04	−0.033	1384	0.009	0.024
Azelaic acid	5.177E−05	5.297E−05	−0.034	1384	0.010	0.025
Docosapentaenoate	3.589E−04	3.532E−04	0.022	1384	0.010	0.025
Erythronic.acid	1.150E−04	1.140E−04	0.012	1384	0.012	0.028
Azelate	3.000E−04	3.112E−04	−0.054	1384	0.014	0.030
Creatine	6.147E−04	5.900E−04	0.057	1384	0.014	0.030
Phytosphingosine	6.802E−05	6.528E−05	0.057	1384	0.014	0.031

Eicosatrienoic acid	1.240E−04	1.187E−04	0.063	1384	0.018	0.038
Lithocholate	1.654E−03	1.785E−03	−0.115	1384	0.019	0.038
N-acetylputrescine	2.393E−04	2.231E−04	0.100	1384	0.022	0.044
Adrenic acid	5.125E−05	4.935E−05	0.054	1384	0.023	0.044
Palmitoyl glycerol	7.182E−05	7.131E−05	0.010	1384	0.023	0.044

Prior to adjusting for covariates, namely age, sex, body mass index (BMI), blood glucose levels, and systolic blood pressure (SBP). [a] Average relative abundance (%) of bacterial species in dyslipidemia case and control. [b] The coefficient and *p*-values were calculated with adjusting for covariates using the generalized linear model implemented in MaAsLin2. The *q*-values were calculated using the Benjamini-Hochberg method for multiple testing correction.

Table S10. Average relative abundance and coefficients of gut metabolite predicted from UniRef90 genes with adjusting for covariates

Metabolites	Relative abundance (%)[a]		MaAsLin2[b]			
	Case	Control	Coefficient	Number of not 0	*p*-value	*q*-value
pseudouridine	4.750E−05	4.838E−05	−0.026	1384	0.007	0.040
uracil	5.853E−04	5.959E−04	−0.026	1384	0.010	0.052
ketodeoxycholate	5.893E−04	5.424E−04	0.100	1384	0.014	0.070
N.acetylhistidine	2.340E−04	2.410E−04	−0.010	1384	0.015	0.071

After adjusting for covariates, namely age, sex, body mass index (BMI), blood glucose levels, and systolic blood pressure (SBP). [a] Average relative abundance (%) of bacterial species in dyslipidemia case and control. [b] The coefficient and *p*-values were calculated with adjusting for covariates using the generalized linear model implemented in MaAsLin2. The *q*-values were calculated using the Benjamini-Hochberg method for multiple testing correction.

Table S11. Top 30 prevalent ARG subtypes in dyslipidemia case and control groups.

Antimicrobial resistance gene (ARG) subtypes	Average abundance (RPKM)[a]			Sample number of not 0
	Case	Control	Total	
tetO	44.985	47.032	45.708	1384
ermB	38.861	37.154	38.258	1382
tetQ	531.901	456.362	505.212	1380
tetW	41.772	48.161	44.029	1378
tet(40)	22.614	24.945	23.438	1377
tet(32)	16.043	16.525	16.213	1377
dfrF	7.639	8.575	7.970	1360
cfxA6	219.783	207.279	215.365	1360
ANT(6)-Ib	19.705	21.665	20.398	1353
ermF	88.777	83.324	86.851	1337
lnuC	6.734	7.904	7.147	1295
ermG	80.060	77.826	79.271	1254
CblA-1	10.949	12.919	11.645	1239
APH(3')-IIIa	1.891	2.239	2.014	1125
tetX	20.678	21.425	20.942	1084
aac(6')-Ie_aph(2")-Ia	4.923	4.886	4.910	1077
EC_acrA	8.719	7.840	8.408	1013
E. coli UhpT	11.435	10.258	11.019	941
CRP	7.141	6.186	6.803	901
SAT4	1.052	1.188	1.100	897
rpoB	11.049	10.033	10.690	881
gadE	5.314	4.634	5.074	873
E. coli fab1	4.528	3.562	4.187	830
tolC	4.731	3.786	4.397	787
E. coli soxS	4.533	3.770	4.264	785
mefA	2.317	1.705	2.101	778
gadX	4.333	5.281	4.668	778
catP	6.571	7.291	6.825	770
arnA	3.563	3.325	3.479	758
E.coli EF-Tu	38.253	30.235	35.420	750

[a] Average abundance (RPKM) of ARG subtypes in dyslipidemia case and control.

Table S12. Internal validation of the association between dyslipidemia and *tetQ* abundance using five repeated 70/30 train-test splits.

Data set	Coefficient	Standard Error	N	N0	*p*-value
Original	0.029	0.010	1384	1380	0.004**
Train #1	0.038	0.012	968	964	0.002**
Test #1	0.009	0.018	416	416	0.607
Train #2	0.021	0.012	968	964	0.083
Test #2	0.047	0.019	416	416	0.012*
Train #3	0.022	0.012	968	965	0.068
Test #3	0.046	0.019	416	415	0.014*
Train #4	0.026	0.012	968	964	0.030*
Test #4	0.043	0.019	416	416	0.025*
Train #5	0.038	0.012	968	966	0.002**
Test #5	0.009	0.019	416	414	0.643

After adjusting for covariates, namely age, sex, body mass index (BMI), blood glucose levels, and systolic blood pressure (SBP). The coefficient and *p*-values were calculated with adjusting for covariates using the generalized linear model implemented in MaAsLin2. *$p < 0.05$, **$p < 0.01$. Abbreviation. N0, Number of not 0

Table S13. Bootstrap-based internal validation of the association between dyslipidemia and *tetQ* abundance.

Mean of coefficient	Median of coefficient	95% CI of coefficient	No. of total bootstrap	No. of direction consistency (beta > 0)	No. of significant effect size ($p < 0.05$)
0.030	0.030	0.010-0.050	1000	997	834

Abbreviation. CI, confidential interval.

Table S14. Correlation analysis of *tetQ* and differently abundant bacterial species

Bacterial species	Correlation coefficient	*p*-value
Coprococcus eutactus	−0.16	< 0.001***
Coprococcus catus	−0.21	< 0.001***
Bacteroides caccae	0.003	0.92
Blautia obeum	−0.20	< 0.001***
Bacteroides stercoris	0.11	< 0.001***
Roseburia inulinivorans	−0.10	< 0.001***
Dorea longicatena	−0.23	< 0.001***

[a] Spearman's correlation test results between relative abundance of *tetQ* and differentially abundant bacterial species in case and control. *$p < 0.05$, **$p < 0.01$.

Table S15. Supplementary analyses testing alternative frequency cutoffs for pork/beef consumption in relation to *tetQ* abundance and dyslipidemia prevalence after adjustment for five covariates.

Exposure variable (meat consumption frequency)	Outcome variable	Model type	*B* (estimate)	SE	*p*-value
Pork consumption more than once per month	*tetQ* abundance	Linear regression	12.38	37.45	0.741
Beef consumption more than once per week	*tetQ* abundance	Linear regression	15.21	30.05	0.613
Pork consumption more than once per month	Dyslipidemia (Case vs. Control)	Logistic regression	−0.37	0.25	0.135
Beef consumption more than once per week	Dyslipidemia (Case vs. Control)	Logistic regression	−0.10	0.19	0.593

All models were adjusted for age, sex, body mass index (BMI), systolic blood pressure (SBP), and blood glucose level. Sample sizes for each consumption category: pork consumption more than once per month (*yes* = 796, *no* = 93) and beef consumption more than once per week (*yes* = 158, *no* = 731).
